# Calcium dynamics at the neural cell primary cilium regulate Hedgehog signaling–dependent neurogenesis in the embryonic neural tube

**DOI:** 10.1073/pnas.2220037120

**Published:** 2023-05-30

**Authors:** Sangwoo Shim, Raman Goyal, Alexios A. Panoutsopoulos, Olga A. Balashova, David Lee, Laura N. Borodinsky

**Affiliations:** ^a^Department of Physiology and Membrane Biology, University of California Davis, Sacramento, CA 95817; ^b^Shriners Hospital for Children, University of California Davis, Sacramento, CA 95817

**Keywords:** Ca^2+^ signaling, sonic hedgehog, primary cilium, neurogenesis, Sox2

## Abstract

The balance between neural stem cell proliferation and neuronal differentiation is paramount for the appropriate development of the nervous system. Sonic hedgehog (Shh) is known to sequentially promote cell proliferation and specification of neuronal phenotypes, but the signaling mechanisms responsible for the developmental switch from mitogenic to neurogenic have remained unclear. Here, we show that Shh enhances Ca^2+^ activity at the neural cell primary cilium of developing *Xenopus laevis* embryos through Ca^2+^ influx via transient receptor potential cation channel subfamily C member 3 (TRPC3) and release from intracellular stores in a developmental stage-dependent manner. This ciliary Ca^2+^ activity in turn antagonizes canonical, proliferative Shh signaling in neural stem cells by down-regulating Sox2 expression and up-regulating expression of neurogenic genes, enabling neuronal differentiation. These discoveries indicate that the Shh-Ca^2+^-dependent switch in neural cell ciliary signaling triggers the switch in Shh action from canonical-mitogenic to neurogenic. The molecular mechanisms identified in this neurogenic signaling axis are potential targets for the treatment of brain tumors and neurodevelopmental disorders.

## Significance

Imbalance between neural cell proliferation and neuronal differentiation during development can result in pediatric cancer or neurodevelopmental disorders. Thus, understanding the mechanisms that control this transition is paramount for preventing and treating these conditions. Here we show that the recruitment of a calcium-dependent mechanism in the developing neural cell primary cilium, converts the developmental signal Sonic hedgehog from proliferative into differentiating. The identified mechanism may become a target for devising therapeutics for brain tumors.

The transition from neural stem cell proliferation to differentiation is crucial for the appropriate development of the nervous system. During neurogenesis, limiting proliferation while promoting differentiation involves extensive cross talk between molecular components of the cell cycle and cell differentiation machinery (1). Spatiotemporal imbalance in this process can be devastating, leading to neurodevelopmental disorders or cancer, but the mechanisms underlying these transitions are not fully understood (2, 3). Sonic hedgehog (Shh) is mostly known for its mitogenic action through canonical Gli transcription factor-dependent signaling, which favors cell proliferation through the upregulation of Cyclin D1 and D2 (4, 5), thus shortening G1 phase length (5). Shh signaling has also been implicated in enabling differentiation of neural progenitors into neurons by up-regulating expression of proneural proteins like Neurogenin 2 (Ngn2) and promoting cell cycle exit (6–8). Thus, Shh signaling regulates both proliferation and differentiation of neural progenitors depending on the cellular context and the presence of neurogenic stimuli, but the precise mechanism that controls these apparently disparate events is still not known.

In vertebrates, Shh signaling has been shown to operate in primary cilia (9, 10), which are microtubule-based sensory organelles that sense the cellular microenvironment and transduce the extracellular signals into cellular responses that regulate cell proliferation and differentiation (11). Disruption of cilia formation and function leads to impaired Shh signaling and complex genetic disorders known as ciliopathies that include defects in nervous system patterning, neural stem cell maintenance, and specification of neural progenitors (12–14).

The transcription factor Sox2 is necessary for maintaining neural stem cell renewal and preventing premature neuronal differentiation (15, 16). Sox2 expression decreases in progenitor cells progressing through neurogenesis as a prerequisite for neuronal differentiation (16–19), but the mechanisms underlying this Sox2 downregulation are unclear. The Shh-Gli canonical signaling axis activates transcription of Sox2 (20, 21), which in turn activates Shh transcription to favor maintenance of neural stem cells (22). Thus, breaking this positive feedback loop may be required for the neurogenic commitment of neural progenitors. Interestingly, Shh-Gli canonical signaling pathway is dramatically down-regulated as neuronal differentiation progresses, despite the persistence of high Shh expression (23–25). Whether there is a ciliary mechanism that leads to Shh-dependent Sox2 downregulation during neuronal differentiation is not known.

Ca^2+^ signaling has been shown to regulate neural stem cell proliferation and neuronal differentiation (26–31), and is even implicated in the earliest developmental stages of neural tube formation (32, 33), but the identity of the ion channels mediating Ca^2+^ signaling in these early stages of neural development is not well defined. Our previous study showed in the developing *Xenopus laevis* spinal cord that inositol-1,4,5-triphosphate (IP3) dynamics are apparent at the developing neuronal primary cilium, and are coordinated with whole-cell Ca^2+^ spikes, which in turn are acutely modulated by Shh signaling (26). Also, Ca^2+^ dynamics were reported at the primary cilium in mouse embryonic fibroblasts and retinal pigmented epithelia cells grown in vitro, as well as in NIH3T3 and mIMCD3 cell lines, all serum-starved to induce ciliogenesis (34, 35), which are mediated by influx through the polycystin 1-like 1 and polycystin 2-like 1 transient receptor potential channel (36). Stimulating Shh signaling by incubating these cells with an agonist of this pathway results in an increase in primary cilium Ca^2+^ levels, but only after 24-h incubation (34). Whether Ca^2+^ dynamics are present at the primary cilium of developing neural cells and what types of mechanisms operate during the transition from neural stem cell to neuron have not been investigated before.

Here, we use a primary cilium-specific ratiometric Ca^2+^ sensor to demonstrate that Ca^2+^ transients are present at the neural cell primary cilium and are acutely activated by Shh signaling in differentiating neurons. We find that TRPC3 protein residing in the primary cilium acts as a key mediator for ciliary Ca^2+^ signaling in response to Shh stimulation. Moreover, inhibition of either TRPC3, Shh, or cAMP signaling enhances Sox2 expression and inhibits neuronal differentiation. We identify downstream molecular targets of Shh-Ca^2+^ signaling at the neural cell primary cilium that promote neuronal differentiation by switching off one of the canonical Shh-Gli target genes during the neural progenitor-to-neuron transition.

## Results

### Primary Cilium Ca^2+^ Dynamics in Neural Cells Are Developmentally Regulated by Shh Signaling and Dependent on Ciliary TRPC3 and IP3 Receptors.

**Fig. 1.**
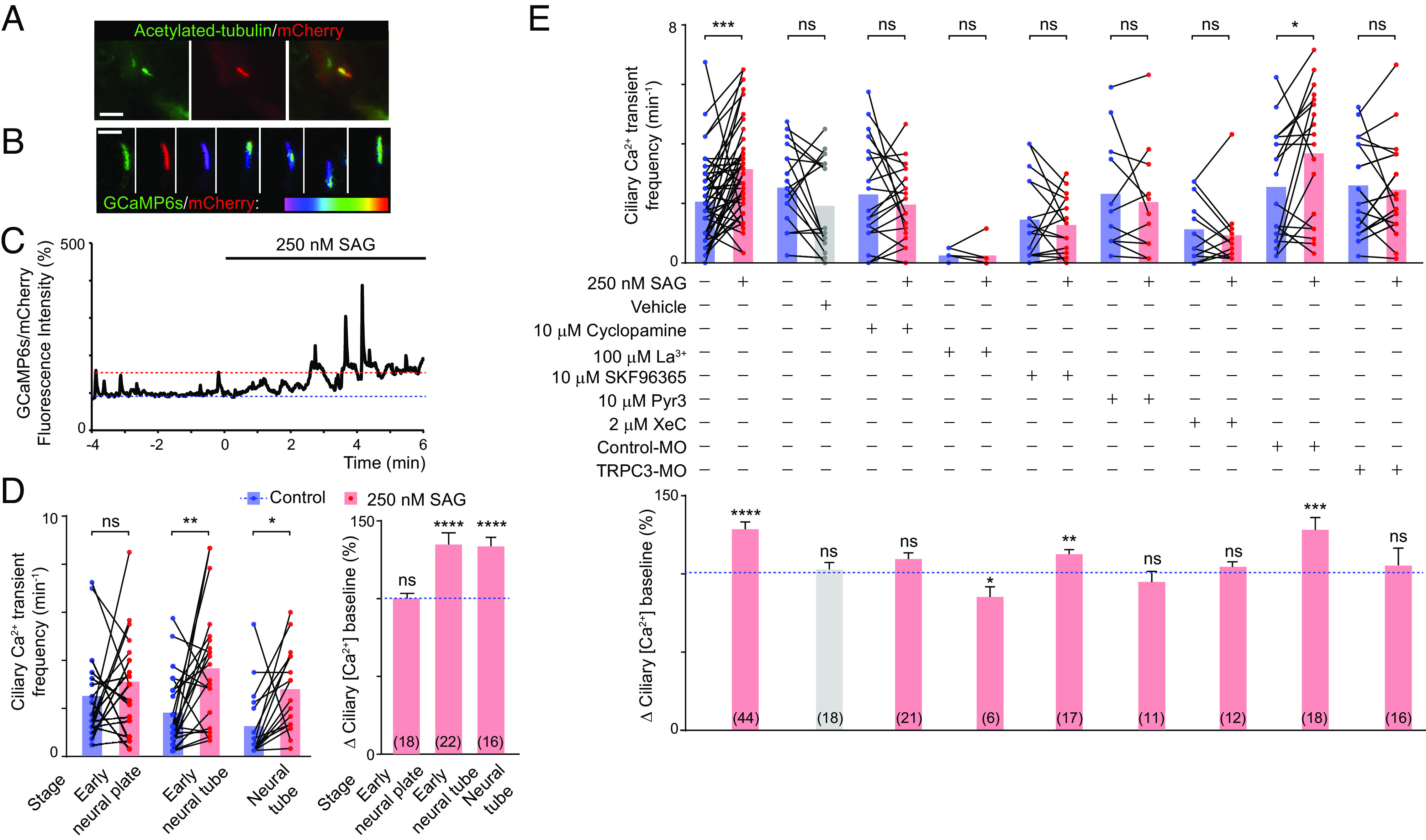
Ca^2+^ dynamics at the neuronal primary cilium are enhanced by Shh and dependent on Ca^2+^ influx through TRPC3 and IP3R-operated Ca^2+^ stores. Dissociated cell cultures from wild-type (*A*–*E*), control-morpholino- (Control-MO) or TRPC3-MO-injected (*E*) *X. laevis* embryo neural tube (*A*–*E*), or neural plate (*D*) expressing the ciliary Ca^2+^ reporter 5HT6-mCherry-GCaMP6s were obtained and either fixed (*A*) or time-lapse imaged for recording GCaMP6s (green) and mCherry (red) fluorescence at the neuronal primary cilium (*B*–*E*) with an acquisition rate of 3.3-5 Hz for a total of 10 min before and after addition of 250 nM SAG (Smo agonist, *C*–*E*). (*A*) Specific localization of 5HT6-mCherry-GCaMP6s Ca^2+^ indicator at the neuronal primary cilia. Immunostaining for mCherry and acetylated tubulin, primary cilia marker, of neuronal cultures from *X. laevis* neural tube (stage 23) expressing 5HT6-mCherry-GCaMP6s. (Scale bar, 10 μm.) (*B*) Images are ratiometric GCaMP6s/mCherry fluorescence in a single neuronal primary cilium at different time points showing transient increases in GCaMP6s fluorescence intensity in different regions of the subcellular structure. White scale bar, 10 μm; colored scale bar, purple: lowest and red: highest GCaMP6s/mCherry fluorescence intensity ratio. (*C*) Example trace of Ca^2+^ activity (GCaMP6s/mCherry fluorescence intensity) at the neuronal primary cilium before (−*x* axis) and after (+*x* axis) addition (0 min) of 250 nM SAG to cultured neurons derived from neural tube (stage 23). Dotted lines represent relative ciliary Ca^2+^ baseline levels reached before (100%, blue) or after (red) SAG addition. (*D*) Shh-induced Ca^2+^ dynamics are developmentally regulated. Neural cell cultures were obtained from early neural plate (stage 14), early neural tube (stage 20), or tailbud spinal cord (stage 28, neural tube). (*E*) Spontaneous and Shh-induced neuronal primary cilium Ca^2+^ transients derived from neural tube (stage 23) are dependent on Ca^2+^ influx and release from stores. In (*D* and *E*), paired data points connected with lines show Ca^2+^ transient frequency for individual primary cilia before and after SAG addition in the absence (*D and E*) or presence (*E*) of indicated agents. Bar graphs show mean±SEM percent change in baseline GCaMP6s/mCherry ratio fluorescence intensity, representing change in baseline ciliary Ca^2+^ concentration, after addition of SAG. In (*D* and *E*) N of cilia analyzed for each group are between parentheses. *P < 0.05, **P < 0.01, ***P < 0.001, ****P < 0.0001, ns: not significant, Wilcoxon matched pairs signed rank test.

Our previous studies and those from others have shown that Shh acutely increases the frequency of Ca^2+^ transients in embryonic spinal cord neuron somas (26) and in retinal ganglion cell growth cones (37). However, whether Shh elicits Ca^2+^ dynamics at the neuronal primary cilium is unknown. In mouse embryonic fibroblasts, stimulation of Shh signaling by SAG, an agonist of the Shh effector Smoothened (Smo), results in an increase in Ca^2+^ levels in the primary cilium, but only after 24-h incubation (34). Here, we investigated how primary cilium-specific Ca^2+^ signaling operates and responds to Shh signaling during neural development. By generating 5HT6-mCherry-GCaMP6s, a primary cilium–targeted ratiometric Ca^2+^ indicator, based on previously engineered Ca^2+^ sensors (34–36), and live imaging of neuronal cultures from neural tube stage *X. laevis* embryos expressing the reporter (Fig. 1*A*), we observed spontaneous Ca^2+^ transients in most neural cell primary cilia (Fig. 1 *B*–*E*). Addition of SAG, thus enhancing Shh signaling, increases both the basal level of Ca^2+^ and the frequency of Ca^2+^ transients at the neuronal primary cilium within few minutes of incubation (Fig. 1 *C–E*). This SAG-stimulated increase in Ca^2+^ transients was prevented by KAAD-cyclopamine, Smo antagonist (Fig. 1*E*), demonstrating acute Smo-mediated Ca^2+^ transients at the neuronal primary cilium.

Simultaneously measuring changes in Ca^2+^ levels in the cell body and in the primary cilium after coexpressing GCaMP6s and 5HT6-mCherry-GCaMP6s (*SI Appendix*, Fig. S1) shows that ciliary and cytoplasmic Ca^2+^ transients can occur synchronously and asynchronously (*SI Appendix*, Fig. S1 *A* and *B* and Movie S1 and S2); there are cytoplasmic Ca^2+^ transients that are also apparent in the primary cilium, while others are not correlated with ciliary Ca^2+^ transients. Similarly, some ciliary Ca^2+^ transients are restricted to that compartment and are not correlated with cytoplasmic Ca^2+^ dynamics (*SI Appendix*, Fig. S1 *A* and *B*). We also find that Shh-stimulated increase in the basal level of Ca^2+^ is only apparent at the primary cilium and not in the cytoplasm (*SI Appendix*, Fig. S1*C*). These results are consistent with previous studies in human retinal pigmented cells and mouse embryonic fibroblasts (34, 38) and indicate that the primary cilium functions as a specialized compartment for Ca^2+^ signaling in response to Shh stimulation in differentiating neurons.

To assess Ca^2+^ dynamics in the neural cell primary cilia at different developmental stages, we prepared dissociated cell cultures derived from the neural plate or neural tube at different embryonic stages, which are enriched in neural stem cells (81 ± 1% Sox2-expressing cells, 0% N-tubulin-expressing cells; early neural plate), neuronal progenitors (25 ± 3% Sox2+, 67 ± 4% N-tubulin-expressing cells with no neuronal morphology; early neural tube) or immature neurons (1 ± 2% Sox2+ cells, 70 ± 10% morphologically distinguishable neurons, neural tube) (23). Interestingly, stimulation of Smo enhances primary cilium Ca^2+^ dynamics only in differentiating neurons, whereas it does not significantly affect ciliary Ca^2+^ level or Ca^2+^ transient frequency of neural stem cells (Fig. 1*D*), suggesting the presence of developmentally and functionally distinct primary cilium-specific Ca^2+^ signaling while transitioning from neural stem cell to neuron.

We then assessed the molecular mechanisms of spontaneous and Shh-induced Ca^2+^ dynamics at the neuronal primary cilium derived from the embryonic neural tube (Fig. 1*E*). We find that primary cilium Ca^2+^ transients are dependent on extracellular Ca^2+^ because incubation of developing neurons with Ca^2+^-free media completely abolished these transients (n = 7 recordings of cultured neurons in Ca^2+^-free media showing no ciliary Ca^2+^ transients). La^3+^, a potent Ca^2+^ channel antagonist, particularly of most TRP channels (39), also resulted in complete inhibition of both spontaneous and SAG-induced Ca^2+^ transients (Fig. 1*E*), suggesting that TRP channels are required for Ca^2+^ activity in the neuronal primary cilium. Additionally, inhibiting Ca^2+^ release from IP3 receptor-operated stores with Xestospongin C (XeC) decreases both spontaneous and SAG-induced ciliary Ca^2+^ dynamics (Fig. 1*E*). Moreover, SKF-96365, an antagonist of store-operated Ca^2+^ entry and TRP channels (40, 41), completely prevents the SAG-induced increase in ciliary Ca^2+^ transient frequency and attenuates the SAG-induced increase in ciliary Ca^2+^ baseline (Fig. 1*E*), suggesting that Ca^2+^ stores and TRP channels participate in Shh-induced Ca^2+^ signaling in the neuronal primary cilium.

**Fig. 2.**
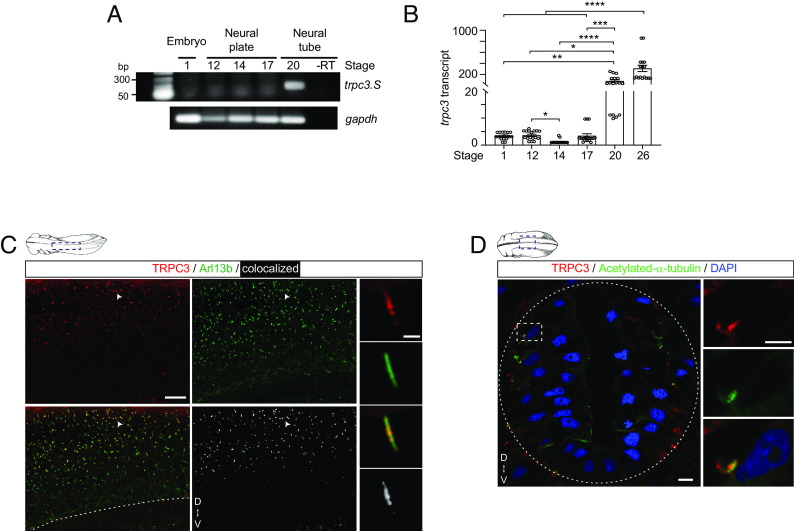
TRPC3 is expressed during primary neurogenesis and localizes to the primary cilium of differentiating neural cells. (*A*) RNA was isolated from embryos at different developmental stages. Reverse Transcriptase-PCR assays for *trpc3*.S, and *gapdh*.S with and without (−) the reverse transcriptase (RT). (*B*) Quantitative RT-PCR was performed in isolated mRNA from different stage embryos for *trpc3.S* and *odc* with the Sybr-green kit for quantitative comparison. Shown are the normalized *trpc3* transcript level compared to the stage with lowest level (assigned value of 1) per experiment. Statistical analysis was performed with the nonparametric Kruskal–Wallis test followed by Dunn’s multiple comparisons test. *P < 0.05, **P < 0.01, ***P < 0.001, ****P < 0.0001. (*C* and *D*) Lateral (*C*) and transverse (*D*) views of representative images of whole-mount (*C*, stage 26) and thin-sectioned (*D*, stage 22) immunostained neural tube for primary ciliary markers Arl13b (*C*), or acetylated-α-tubulin (*D*), and TRPC3 (*C* and *D*). (*C*) Zoomed-out image is a maximum intensity projection (20× objective, 40 optical frames). Arrowheads indicate example of the TRPC3-immunolabeled primary cilium shown in zoomed-in images corresponding to a single optical frame. The dashed line indicates the ventral border of the scanned neural tissue. (*D*) Image shown is a single optical frame. The dashed box indicates zoomed-in region and dashed oval contours the transverse section of the neural tube. [Scale bars in *C*, 20 and 5 μm (in magnified images) and in *D*, 10 and 5 μm (in magnified images).] D, dorsal; V, ventral.

Because TRPC3 has been shown to localize primarily in the apical membrane of polarized kidney and epithelial cells (42) where primary cilium assembly takes place (43), we investigated whether TRPC3 is expressed and localizes to primary cilia of neural cells in developing embryos. We find that the mRNA expression of *trpc3.S*, the dominantly expressed homeolog of *X. laevis trpc3* based on RNAseq data (44), increases as neural tube formation progresses (Fig. 2 *A* and *B*). Moreover, TRPC3 protein colocalizes with ciliary markers Arl13b (Fig. 2*C*; 35 ± 5% Arl13b+ cilia are TRPC3+, mean±SEM, n = 8 neural tubes) and acetylated α-tubulin (Fig. 2*D*) in the developing neural tube. These results suggest that TRPC3 is suitably positioned to effectively enable primary cilium-specific Ca^2+^ signaling during neural development. Also, addition of TRPC3 agonist, GSK 1702934A, increases Ca^2+^ transient frequency and Ca^2+^ baseline levels at the primary cilium and not in the cytoplasm (*SI Appendix*, Fig. S1 *D* and *E*), further supporting functional ciliary localization of TRPC3. To assess the role of TRPC3 on ciliary Ca^2+^ dynamics, we pharmacologically inhibited the activity of TRPC3 with its selective antagonist Pyr3 (45), and find that it decreases SAG-induced Ca^2+^ activity in the neuronal primary cilium (Fig. 1*E*). Moreover, knocking down *trpc3* expression by TRPC3-specific morpholino (TRPC3-MO) that interferes with *trpc3*.S splicing, thus depleting *trpc3* mature mRNA and TRPC3 protein levels (*SI Appendix*, Fig. S2), prevents the SAG-induced increase in neuronal ciliary Ca^2+^ activity compared with Control-MO samples, mimicking the effect of pharmacologically inhibiting TRPC3 (Fig. 1*E*). Altogether these results suggest that both Ca^2+^ influx through TRPC3 and Ca^2+^ release from intracellular stores participate in Shh-modulated ciliary Ca^2+^ dynamics in developing neurons.

### TRPC3 Is Necessary for Neurogenesis in the Developing Neural Tube.

**Fig. 3.**
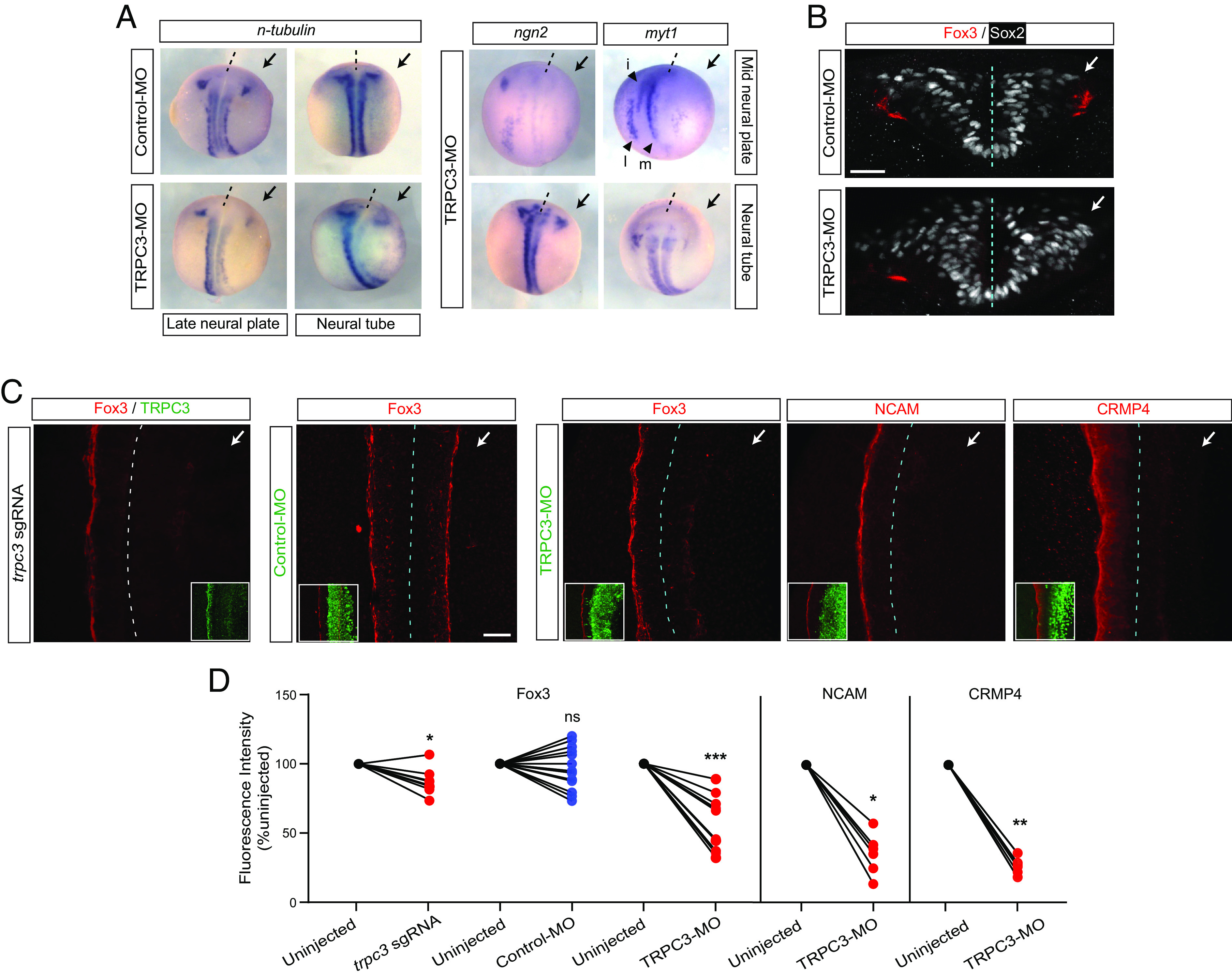
TRPC3 is necessary for embryonic neurogenesis. Two-cell stage embryos were unilaterally injected with TRPC3- or Standard Control-Morpholino [TRPC3-MO or Control-MO, (*A*–*D*)] or with Cas9/*trpc3*-specific sgRNA (*C* and *D*), along with rhodamine-dextran tracer (*A*) or GFP mRNA (*B*–*D*), and processed for in situ hybridization (*A*) or whole-mount immunostaining (*B*–*D*) when they reached mid (stage 15) or late (stage 19) neural plate, or early neural tube (stage 22) stages. (*A*) Representative images of dorsal view from whole-mount in situ hybridization for neuronal markers *n-tubulin* and *myt1*, and proneural marker *ngn2*. Three stripes of primary neurons (medial, m; intermediate, i; lateral, l) are indicated with arrowheads at the uninjected side. Dashed line indicates midline, anterior is up; injected side marked by black arrow. (*B* and *C*) Representative transverse (*B*) or dorsal (*C*) views of images of whole-mount immunostained neural tubes (stage 22) for neuronal markers Fox3, NCAM, and CRMP4. Dashed line indicates neural tube midline; injected side marked by white arrow. [Scale bars, 30 (*B*) and 50 (*C*) μm.] In *C*, *Insets* show decreased TRPC3 expression in *trpc3*-sgRNA-injected side (most left image), or side expressing injected tracer (GFP immunostaining in green) along with MOs (four most right images). (*D*) Data points are relative fluorescence intensities for the indicated neuronal marker as the ratio of mean percent intensity in injected compared with uninjected side in individual embryos. N ≥ 13 embryos for Fox3, N = 9 embryos for NCAM and N = 6 for CRMP4. *P < 0.05, **P < 0.01, ***P < 0.001, ns: nonsignificant, Wilcoxon matched pairs signed rank test.

To assess the potential function of TRPC3 in neurogenesis, we knocked down TRPC3 expression (*SI Appendix*, Fig. S2) by injecting TRPC3-MO unilaterally in 2-cell stage embryos, and find lower levels of transcripts for neuronal marker *n-tubulin*, neuronal basic helix–loop–helix transcription factor *ngn2,* and early neuronal marker myelin transcription factor 1 (*myt1*) in TRPC3-deficient neural tissue compared to control (Fig. 3*A*). All three stripes of primary neuron progenitors, medial (motor neuron), intermediate (interneuron), and lateral (sensory neuron) in the neural plate are reduced, and this reduced neuronal marker expression persists at later neural tube stages (Fig. 3*A*). TRPC3 knockdown by TRPC3-MO (*SI Appendix,* Fig. S2) or by Crispr-Cas9-trpc3-specific sgRNA that yields a high allelic indel frequency and knockout score for *trpc3*.S (*SI Appendix*, Fig. S3) preventing expression of TRPC3 protein (Fig. 3*C*), also reduces neuronal marker Forkhead box 3 (Fox3) expression in the embryonic neural tube, while Control-MO had no effect on Fox3 expression levels (Fig. 3 *B*–*D*). Moreover, expression of additional neuronal markers, Neural Cell Adhesion Molecule (NCAM) and Collapsin Response Mediator Protein 4 (CRMP4) is reduced in the TRPC3-deficient half of the neural tube compared with the wild-type counterpart (Fig. 3 *C* and *D*). Altogether these results suggest that TRPC3 is required for neuronal differentiation by promoting expression of neurogenic genes.

### Sox2 Is a Downstream Target of Shh-TRPC3 Signaling during the Neural Progenitor-to-Neuron Transition.

**Fig. 4.**
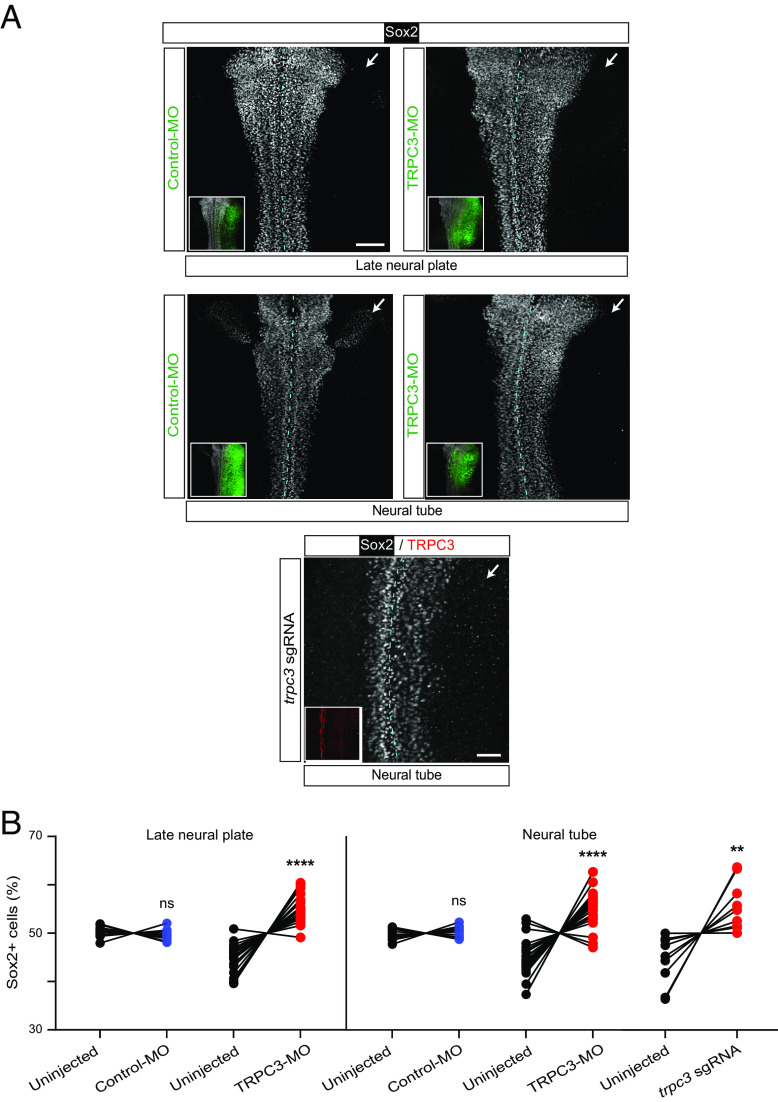
TRPC3 knockdown increases the number of Sox2-expressing neural stem cells. Two-cell stage embryos were unilaterally injected with TRPC3-morpholino (TRPC3-MO), standard control-morpholino (Control-MO) along with GFP mRNA, or with Cas9/*trpc3* sgRNA and processed for whole-mount immunostaining at late neural plate (stage 18) and early neural tube (stage 22) stages. (*A*) Images are representative dorsal view of whole-mount immunostained samples for neural stem cell marker Sox2. *Insets* indicate injected side by immunostaining for GFP or TRPC3. Dashed line indicates midline. [Scale bars, 100 (*Top*/*Middle*) or 50 (*Bottom*) µm.] (*B*) Graph shows percent of number of Sox2-expressing neural stem cells in injected and uninjected sides of neural tissue in individual embryos. Lines connect paired sides of neural tissue from individual embryos. N ≥ 13 (Control-MO), ≥19 (TRPC3-MO) and ≥9 (*trpc3*-sgRNA) embryos, **P < 0.01, ****P < 0.0001, ns: nonsignificant, Wilcoxon matched pairs signed rank test.

The inhibition of neurogenesis could be due to either interference with generation of neuroepithelial cells, namely neural induction, or subsequent neuronal differentiation from these cells. To distinguish between these possibilities, we performed whole-mount immunostaining for the neural stem cell marker Sox2 in embryos unilaterally injected with TRPC3-MO or Control-MO. We find that TRPC3 knockdown (*SI Appendix*, Fig. S2) increases the number of Sox2-expressing cells in the developing neural plate and neural tube compared with the uninjected side, while Control-MO did not affect the number of neural stem cells in either of these stages (Fig. 4 *A* and *B*). Similarly, down-regulating TRPC3 function by the Crispr/Cas9 gene editing approach (*SI Appendix*, Fig. S3) resulted in significant expansion of the Sox2-expressing neural tube domain and increase in Sox2-expressing cells in the neural tube (Fig. 4 *A* and *B*), mimicking the effect of TRPC3-MO-driven knockdown. These results suggest that TRPC3 knockdown-mediated inhibition of neurogenesis is due to the failure of neural stem cells to down-regulate Sox2 expression and differentiate into neurons.

**Fig. 5.**
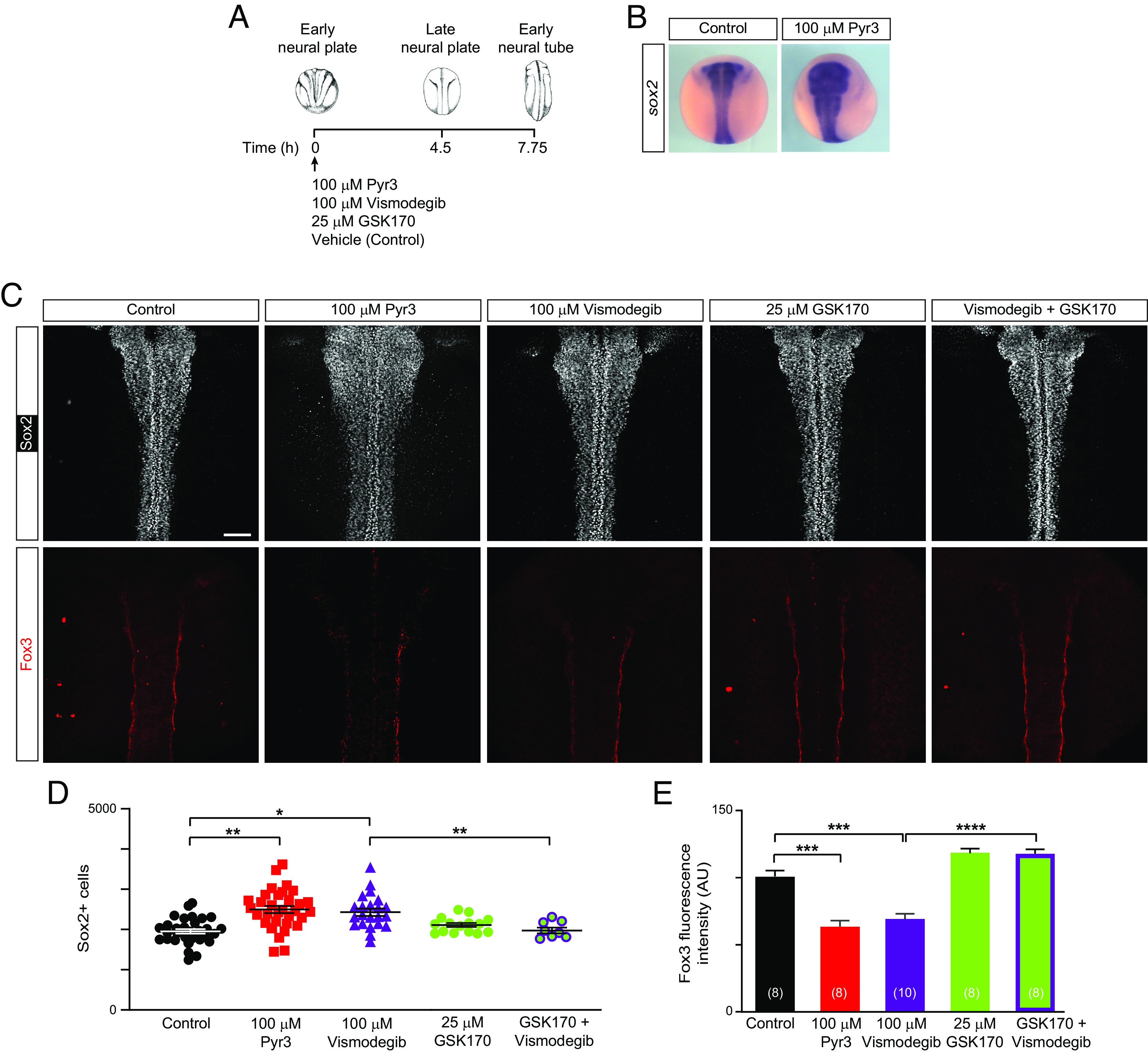
Inhibition of TRPC3 and Shh signaling during the neural progenitor-to-neuron transition inhibits neuronal differentiation. Wild-type embryos were grown until early neural plate stage (stage 14) when they were incubated with 100 μM Pyr3 (TRPC3 inhibitor), 100 μM vismodegib, (Smo inhibitor), 25 μM GSK1702934A (GSK170, TRPC3 agonist), a mix of vismodegib and GSK170, or vehicle only (0.1% DMSO, Control) for 4.5 h until they reached the late neural plate stages (stage 19, *B*) or for 7.75 h until they reached early neural tube stages (stage 22, *C*-*E*), when they were processed for whole-mount in situ hybridization (*B*) or whole-mount immunostaining (*C*–*E*). (*A*) Diagram of embryo treatment during the neural progenitor-to-neuron transition. (*B*) Representative images of whole-mount in situ hybridization for the neural stem cell marker *sox2*. (*C*) Representative dorsal view of whole-mount immunostained neural tubes for neural stem cell marker Sox2 and neuronal marker Fox3. (Scale bar, 100 µm.) (*D*) Graph shows number of Sox2-expressing cells per embryo analyzed and mean±SEM. *P < 0.05, **P < 0.01, Mann–Whitney*** U-test. (*E*) Graph shows mean ± SEM fluorescence intensity of Fox3 immunolabeling, N of embryos indicated in parentheses, *P < 0.05, Mann–Whitney U-test.

To assess the effect of specific inhibition of TRPC3 during the neural progenitor-to-neuron transition on neurogenesis progression, we incubated embryos with TRPC3 inhibitor Pyr3 from neural plate through early neural tube stages (Fig. 5*A*). We find that inhibiting TRPC3 pharmacologically during this period of peak neurogenesis is sufficient to cause an expansion of the neural stem cell, *sox2*-expressing, region in the neural tissue compared with controls (Fig. 5*B*). Results also show that inhibiting TRPC3 during the neural progenitor-to-neuron transition increases the number of Sox2 protein-expressing neural stem cells (Fig. 5 *C* and *D*) in a concentration-dependent manner (*SI Appendix*, Fig. S4), and decreases Fox3 (Fig. 5 *C* and *E*) and CRMP4 (*SI Appendix*, Fig. S5) expression in the recently formed neural tube compared with control embryos. Altogether these results demonstrate that TRPC3 enables the differentiation of neural stem cells into neurons likely by down-regulating Sox2 expression.

Since we identified that TRPC3 is a key mediator of Shh-induced Ca^2+^ signaling at the neural cell primary cilia, we next investigated whether inhibition of Shh-Smo signaling recapitulates loss of neurogenesis by TRPC3 knockdown. Inhibiting Shh-Smo signaling during the neural progenitor-to-neuron transition by treating embryos with Smo inhibitor vismodegib from neural plate through early neural tube stages (Fig. 5*A*) increases the number of Sox2-expressing neural stem cells (Fig. 5 *C* and *D*) and the level of Sox2 protein (*SI Appendix*, Fig. S6), and decreases expression of neuronal markers Fox3 (Fig. 5 *C*–*E*) and CRMP4 (*SI Appendix*, Fig. S5), mimicking TRPC3 inhibition-induced phenotypes. Moreover, activating TRPC3 with a specific agonist, GSK 1702934A (GSK170) while inhibiting Shh-Smo signaling during primary neurogenesis rescues the vismodegib-induced phenotype by restoring the number of Sox2-expressing cells (Fig. 5 *C* and *D*) and Sox2 protein levels (*SI Appendix*, Fig. S6), as well as expression of Fox3 (Fig. 5 *C* and *E*) in the developing neural tube, to control values. These results indicate that Shh signaling is necessary for the transition from neural stem cell to neuron during the neurogenic period and suggest that TRPC3 acts as downstream mediator of Shh signaling in the regulation of neurogenesis.

### Adenylate Cyclase Activity Is Necessary for Neurogenesis.

**Fig. 6.**
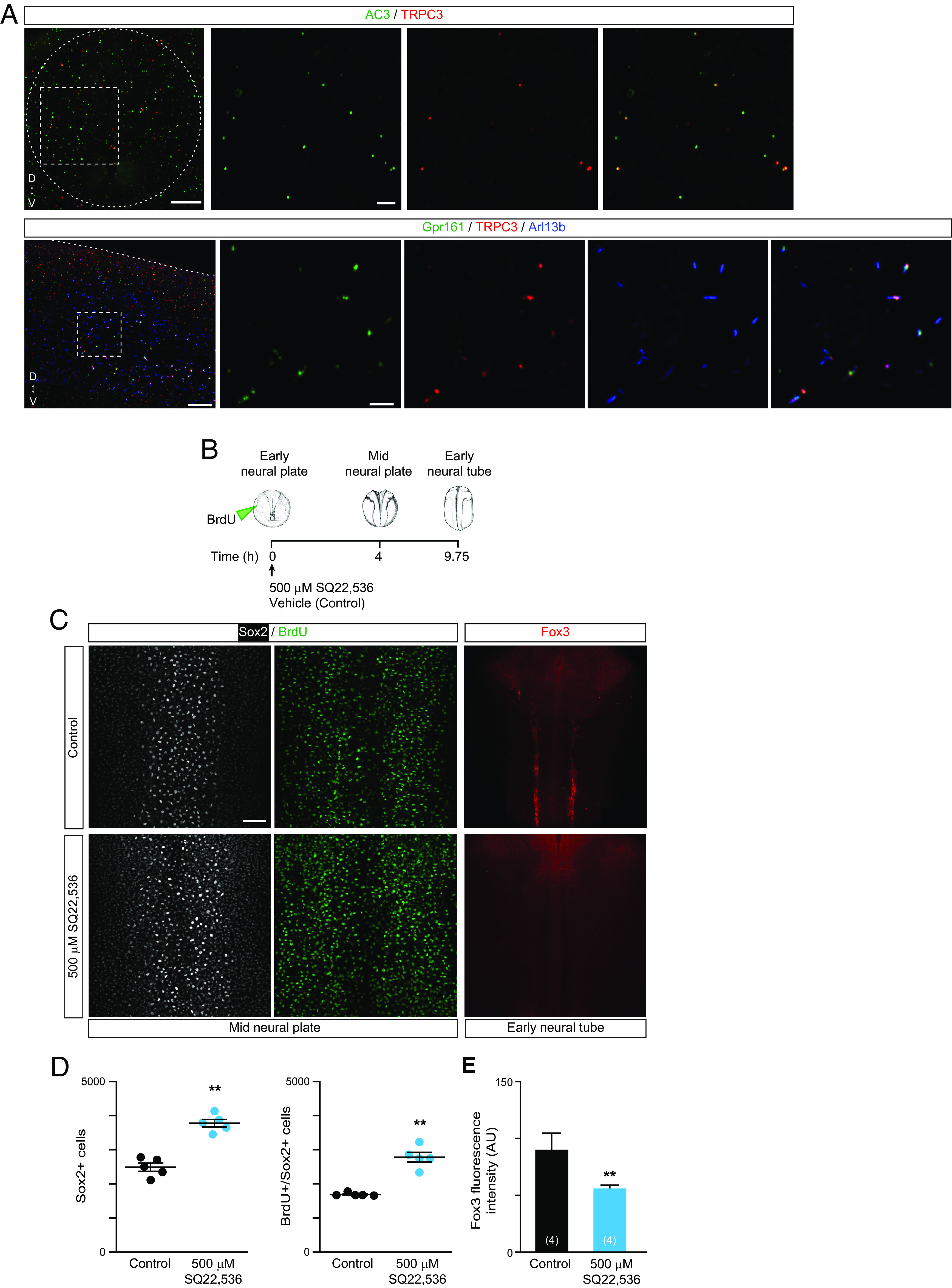
Sox2 expression and neural cell proliferation are regulated during neurogenesis in an adenylate cyclase-dependent manner. (*A*) Representative images of transverse (*Upper*) and lateral (*Bottom*) views of whole-mount early neural tube (stage 22) immunostained for adenylate cyclase 3 (AC3) and TRPC3, and mid neural tube (stage 25) immunostained for Gpr161 (ciliary GPCR), TRPC3, and primary ciliary marker Arl13b. Dashed box indicates zoomed-in region. Dashed oval contours the transverse section of the neural tube and dashed line indicates the dorsal border of the scanned neural tissue. Zoomed-out images are a maximum intensity projection (20× objective, 40 optical frames). Zoomed-in images are single optical frames. (Scale bars are 20 and 5 μm in zoomed-out and zoomed-in images, respectively.) (*B*–*E*) Wild-type embryos were grown until early neural plate stage (stage 12.5) when they were microinjected with 10 μM BrdU into the blastocoel cavity and incubated with 500 μM SQ22,536, adenylate cyclase inhibitor, or vehicle only (0.5% DMSO, Control) until midneural plate (stage 16) or early neural tube (stage 22) stages for whole-mount immunostaining. (*B*) Diagram of treatments during the neurogenic transition. (*C*) Representative dorsal view of whole-mount-immunostained neural plate for neural stem cell marker Sox2 and incorporated BrdU at the indicated developmental stages. (Scale bar, 50 μm.) (*D*) Graphs show number of Sox2+ and BrdU+/Sox2+ cells per embryo analyzed and mean ± SEM. **P < 0.01, compared to Control, Mann–Whitney U-test. (*E*) Graph shows mean ± SEM fluorescence intensity of Fox3 immunolabeling, N of embryos indicated in parentheses, **P < 0.01, Mann–Whitney U-test.

The cAMP-activated kinase, PKA, serves as a potent negative regulator of canonical Shh signaling pathway through cilia-dependent processing of Gli into transcriptional repressor (23). Gpr161 is a G protein-coupled receptor that localizes to the primary cilium and antagonizes canonical Shh-mediated signaling in the neural tube by activation of adenylate cyclase, elevation of cAMP, and activation of PKA (46). PKA functionally localizes to the primary cilium (47, 48) and ciliary PKA activity, which is specifically regulated by ciliary cAMP, regulates Gli transcription factors (48). Interestingly, Gpr161 has been shown to anchor PKA signalosome to primary cilia in complexes that include TRPC3 (49). Hence, we examined whether the regulation of neural stem cell-to-neuron transition is dependent on cAMP signaling. We find that adenylate cyclase 3 (AC3), known to localize to primary cilia, partially colocalizes with TRPC3 (Fig. 6*A*; 30 ± 1.5% AC3+ cilia are TRPC3+, mean ± SEM, n = 5 neural tubes) and with Arl13b (*SI Appendix*, Fig. S7) in the developing neural tube. We also find that Gpr161 colocalizes with TRPC3 in the primary cilia of neural cells in the neural tube (Fig. 6*A*; 38 ± 5% Gpr161+ cilia are TRPC3+, mean ± SEM, n = 7 neural tubes). Inhibiting adenylate cyclase activity during the neurogenic transition by incubating embryos with the inhibitor SQ22,536 (Fig. 6*B*) promotes proliferation of neural stem cells (BrdU+/Sox2+) and increases the number of Sox2-expressing cells in the neural plate (Fig. 6 *C* and *D*). In contrast, inhibiting adenylate cyclase reduces neuronal differentiation as revealed by decreased Fox3 expression in early neural tube stages compared with controls (Fig. 6 *C* and *E*). Hence, reduced neurogenesis by inhibiting cAMP signaling correlates with increased Sox2 expression and maintenace of the proliferative status of neural stem cells.

Altogether, these results suggest that adenylate cyclase-cAMP-PKA signaling in the primary cilia of neural progenitors may mediate Shh-TRPC3-Ca^2+^-dependent neurogenesis through the downregulation of Sox2 expression.

## Discussion

Shh signaling in the primary cilium regulates both proliferation and differentiation of neural progenitors depending on the cellular context, but the precise ciliary signaling mechanism leading to regulation of neuronal differentiation has remained unclear. This study shows that Shh enhances Ca^2+^ activity at the neural cell primary cilium by recruiting Ca^2+^ influx through TRPC3 and release from IP3 receptor-operated stores. This ciliary Ca^2+^ signaling, in turn is necessary for down-regulating Sox2 expression, presumably, in a cAMP-dependent manner to promote neuronal differentiation. Thus, we report a previously unidentified mechanism through which Shh-Ca^2+^ signaling axis in the primary cilium acts as a critical enabler of neurogenesis.

Advances in the understanding of primary cilium-specific signaling have been made mainly through studies using serum-starved, immortalized fibroblast cell lines to allow for cell arrest and primary ciliogenesis. This study argues that signaling rules established in these cells may not be shared among other cell types and cell cycle phases. Moreover, neural cells, in particular neurons, are highly specialized with their own distinctive proteome, especially ion channels, which differ from fibroblasts and other cell lines. Using a primary cilium-specific ratiometric Ca^2+^ sensor, we show that, in differentiating neurons, Shh stimulation acutely increases Ca^2+^ transients in this organelle through the TRPC3 channel, unlike in fibroblasts where ciliary Ca^2+^ elevations through PKD2L1 are only apparent after 24-h incubation with SAG (34). We also find that Shh signaling enhancement of ciliary Ca^2+^ activity is exclusive of differentiating neurons and not apparent in neural stem cells. These findings support the notion that the primary cilium is a functionally distinct subcellular Ca^2+^ compartment operating in a cell type- and developmental-stage-specific manner. Indeed, a recently published study shows that rodent brainstem serotonergic axons establish synapses with the primary cilium of hippocampal CA1 pyramidal neurons (50), demonstrating highly specialized signaling in the neuronal primary cilia. Compartmentalization of signaling pathways at the primary cilium is also apparent in the cilium-generated cAMP that inhibits canonical Hh transduction while cytoplasmic cAMP does not (48).

**Fig. 7.**
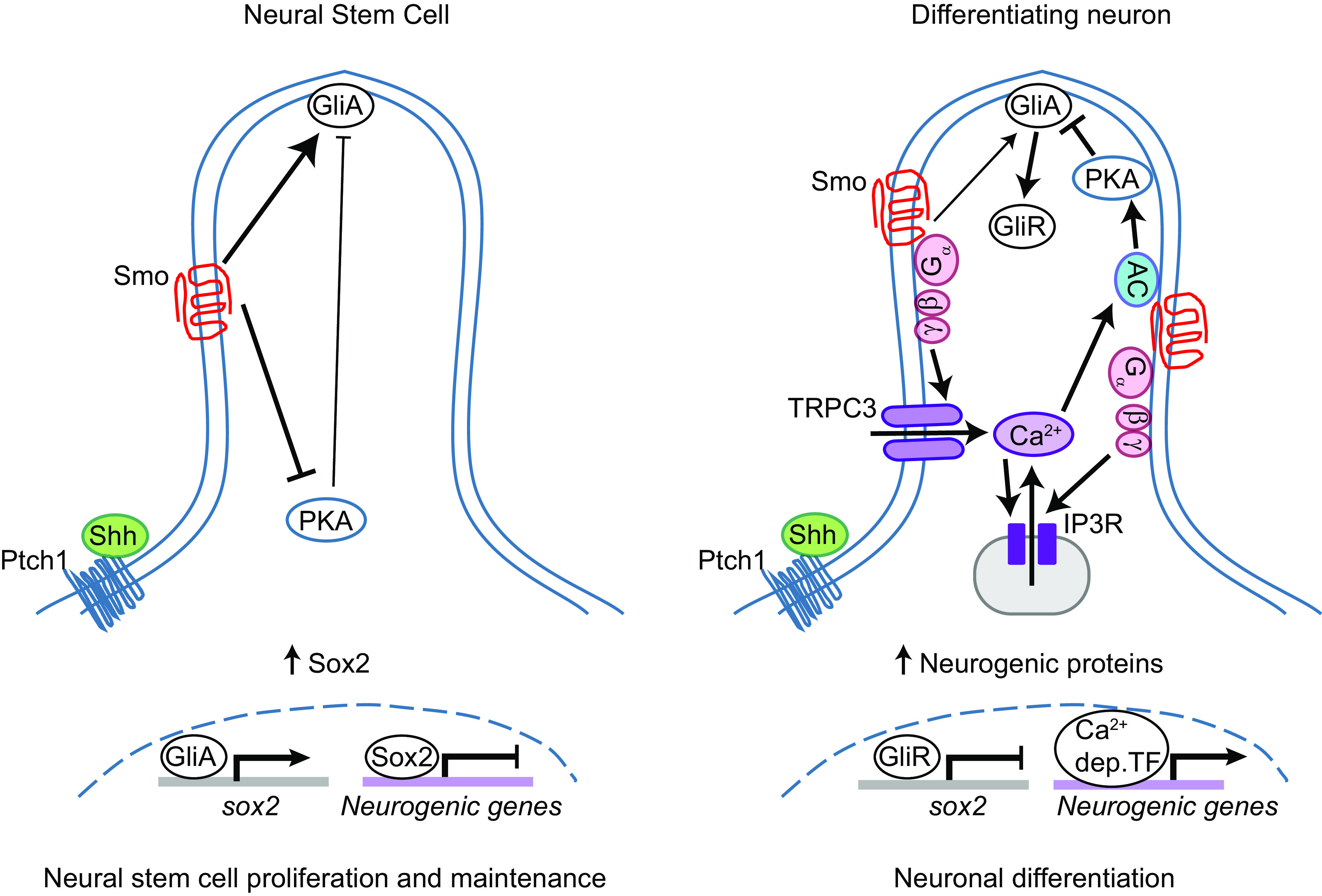
Model of Shh-mediated ciliary Ca^2+^/cAMP signaling underlying the neural progenitor-to-neuron transition. Differentiating neurons exhibit a primary cilium where Shh coupled with TRPC3 and IP3-regulated stores generate ciliary Ca^2+^ dynamics that recruit cAMP signaling to inhibit canonical, proliferative Shh signaling in neural stem cells by down-regulating Sox2 expression and up-regulating expression of neurogenic genes, thus enabling neuronal differentiation. GliA: Gli activator, GliR: Gli repressor, AC: adenylate cyclase, IP3R: IP3 receptor, Ca^2+^ dep TF: Ca^2+^-dependent transcription factors.

A role of Ca^2+^ dynamics in mediating Shh-induced cellular responses has been recognized by several lines of investigation. Ryanodine receptor–mediated intracellular Ca^2+^ mobilization regulates the level of Shh-dependent gene expression and cell specification in the somitic muscle and neural tube of zebrafish embryos (51). Shh signaling also activates the expression of Connexin-43, a member of the gap junction family, which along with Ca^2+^ release-activated channels and voltage-gated Ca^2+^ channels contributes to synchronized Ca^2+^ oscillations, thereby coordinating cell migration patterns during chicken feather bud elongation (52). Our previous study showed that Shh-mediated whole-cell Ca^2+^ spike activity induces an inversion of Gli transcriptional activity from activator to repressor through activation of PKA to regulate neuron specification in the developing spinal cord (23, 26). This study identifies TRPC3 as a Ca^2+^ channel present at the primary cilium of differentiating neurons and modulated by Shh signaling. Previously proposed mechanisms of TRPC3 activation include Ca^2+^ release from IP3 receptor (IP3R)-operated Ca^2+^ stores (53, 54) and binding by diacylglycerol (55), which can be produced by G-protein-recruited signaling (Fig. 7). Our previous study showed localization of IP3R at the base of the primary cilium, and Shh-dependent localization of IP3 transients at the primary cilium preceding the onset of Ca^2+^ spikes (26), suggesting that Shh-induced Ca^2+^ spikes depend on IP3-induced Ca^2+^ release from intracellular stores. In the present study, we show the localization of TRPC3 to primary cilia in the developing neural tube, that, together with IP3R-expressing Ca^2+^ stores at the base of the primary cilium (26), suggest a ciliary functional localization of store-operated Ca^2+^ entry components for Shh-specific signaling. Taken together, we propose a model for store-operated TRPC3 activation at primary cilia of differentiating neurons. Shh activates Smo at the primary cilium, resulting in the recruitment of G proteins, activation of PLC, and increase in IP_3_ and diacylglycerol levels. Opening of IP3R-operated stores triggers Ca^2+^ release from nearby stores, which in turn leads to Ca^2+^ influx through TRPC3 (Fig. 7). Spatiotemporal expression and localization of signaling molecules in neural cells transitioning out of the stem cell status result in Shh-Ca^2+^-mediated neuronal differentiation. In this regard, the primary cilium of neural progenitor cells is well positioned and equipped to function as an effective signaling compartment for this noncanonical Shh-Ca^2+^ pathway.

This noncanonical Shh signaling mechanism that the present study identifies at the neural cell primary cilium appears to promote the switch in Shh function from the well-recognized proliferative role to a less appreciated, yet as important role in cell differentiation. Shh signaling induces neural progenitor cell proliferation by increasing Cyclin D1 expression and shortening G1 phase length (5), but also promotes cell differentiation by regulating transcription of factors and proteins that drive cell cycle exit (6–8), thus, antagonizing its own canonical, Gli activator-dependent, Shh signaling. Interestingly, Shh-Gli canonical signaling pathway is dramatically down-regulated as neuronal differentiation progresses, despite the persistence of Shh expression (23–25). Moreover, we previously discovered that in the developing neural tube Shh-mediated Ca^2+^ activity contributes to the shutting off of the canonical Gli-dependent Shh signaling pathway by recruiting PKA, which in turn promotes the conversion of Gli activators into repressors, impedes the shuttling of Gli activators to the nucleus, and represses Gli1 transcription by activating CREB transcription factor (23). Here, we show that Shh-dependent TRPC3/IP3R-mediated ciliary Ca^2+^ activity is likely the trigger of the switch in Shh function from mitogenic into neurogenic.

We identified Sox2 as a downstream target of Shh-ciliary Ca^2+^ signaling axis in the transition from neural stem cell to neuron. Proneural basic helix–loop–helix factors such as Ngn2 are also required for driving neuronal differentiation by activating a cascade of proneuronal genes and repressing Sox2 expression, which results in cell cycle exit and differentiation of neural progenitors (17). In this study, we demonstrate that ciliary Shh-TRPC3 signaling axis is necessary for down-regulating Sox2 expression concomitant with upregulation of Ngn2 expression. This downregulation of Sox2 by ciliary Shh-TRPC3-calcium signaling may be due to either repression of its transcription by Gli repressor (20), or indirectly through upregulation of Ngn2 (17). Thus, the present study provides a mechanism by which Ca^2+^ signaling at the primary cilium acts as a key determinant for Shh-mediated proliferation vs differentiation decisions through repressing Sox2 expression.

This study further identifies cAMP as part of the signaling pathway in the neural stem cell-to-neuron transition during embryonic development. Primary cilium adenylate cyclase is a known inhibitor of the canonical Gli-dependent Shh signaling by increasing cAMP levels, which activate PKA to promote the processing of Gli transcription factors into repressors (56, 57). Changes in Ca^2+^ signaling can regulate cAMP levels and PKA activity through the modulation of different isoforms of Ca^2+^-sensitive adenylate cyclase. Remarkably, while enhancing Shh signaling in early neural plate inhibits PKA activity, enhancing Shh signaling in the developing neural tube increases PKA activity in a Ca^2+^ spike activity-dependent manner (23). This is consistent with the upregulation of cAMP-generating activity of ciliary G-protein-coupled receptors by Hh signaling in mIMCD3 cells (58). We find a Ca^2+^-activated adenylate cyclase, AC3, and Gpr161, known to increase cAMP levels, expressed in the developing neural tube and localized to the neural cell primary cilium. Moreover, interactomic data identified Gpr161 as an anchoring protein of PKA signalosome at the primary cilium that includes TRPC3 (49). Inhibiting adenylate cyclase activity impedes neuronal differentiation by increasing neural stem cell proliferation, mimicking the phenotype induced by loss of Shh or TRPC3 function during the period of primary neurogenesis. Given that we find that Shh enhances Ca^2+^ activity in the neuronal primary cilium and that a Ca^2+^-dependent adenylate cyclase localizes to this structure, our model proposes that developmentally regulated recruitment of the Shh-Ca^2+^-cAMP signaling at the neural cell primary cilium shifts Shh action from proliferative to neurogenic (Fig. 7). Further investigation is needed to determine the actual recruitment of PKA activity by the ciliary Shh-TRPC3-Ca^2+^ signaling responsible for neurogenesis.

Dysregulations in Shh signaling and ciliogenesis are implicated in brain tumors, such as medulloblastoma (MB) (59–61). Perturbed neurogenesis is associated with MB formation (62), but the molecular switches that block normal neuronal differentiation are unclear. Interestingly, a recent study shows that TRPC3 is consistently down-regulated in three independent human MB datasets including all the 4 MB subgroups compared to normal brain tissue, and in spontaneous MB mouse models (63). Endoplasmic reticulum Ca^2+^ regulators, ITPR1 (IP3R type I), RYR1, RYR2, and STIM1 were also significantly down-regulated in MB tissues. Moreover, dysregulation of Sox2 expression has been implicated in the pathogenesis of MB (21, 64–66). Thus, our identification of ciliary TRPC3 and Ca^2+^ stores as key players in regulating Shh-mediated Sox2 expression and neuronal differentiation may provide an insight into the pathological mechanisms and therapeutic strategy for MB and other diseases.

## Materials and Methods

### Animals.

Freshly laid *Xenopus laevis* eggs were fertilized in vitro with dissected testis in 10% Marc’s Modified Ringer (MMR) solution [10 mM NaCl, 0.2 mM KCl, 0.1mM MgSO_4_, 0.2 mM CaCl_2_, 0.5 mM HEPES (pH 7.8), 0.01 mM EDTA]. Animals were raised at room temperature in 10% MMR until the stage required for each experiment. Animal handling was performed according to IACUC regulations and under an approved animal protocol.

### Neural Cell Culture.

*Xenopus* neural plate or spinal cord cells were dissociated from stage 14 to 28 embryos and cultured for 4 h to overnight as previously described (67), in a saline solution (in mM): 117 NaCl, 2 CaCl_2_, 1.3 MgCl_2_, 0.7 KCl, 4.6 Tris-Base, pH 7.8.

### Expression of Cytosolic and Primary Cilium–Targeted Ca^2+^ Sensor.

A ciliary-targeted ratiometric Ca^2+^ sensor, 5HT6-mCherry-GCaMP6s was generated by fusing GCaMP6s (Plasmid #40753, Addgene) to the 5HT6-mCherry (derived from Plasmid #47500, Addgene) in pCS2 expression vector. Cytosolic Ca^2+^ sensor used was generated from pCS2-GCaMP6s construct. Full-length serotonin receptor 5HT6 target the construct preferentially to the primary cilium, and the mCherry fluorescent marker normalizes the potential spatial movement of the cilium. mRNAs encoding GCaMP6s and 5HT6-mCherry-GCaMP6s were obtained by in vitro transcription (mMessage mMachine SP6 kit, Ambion) from the linearized template. Two hundred pg of mRNA was injected per blastomere in 2-cell stage embryos.

### Primary Cilium Ca^2+^ Imaging.

Neural cell cultures were time-lapse imaged under a Sweptfield confocal microscope (Nikon) with 488- and 568-nm lasers at 3.3 to 5-Hz acquisition rate over 10 min before and after addition of 250 nM SAG (Calbiochem). To analyze the ciliary Ca^2+^ transients, the dual fluorescence intensities of 5HT6-mCherry-GCaMP6 in the cilium were first background subtracted using the fluorescence intensity of an adjacent cell-free region, and a region of interest (ROI) was demarcated for the primary cilium at each time point using the tracking module of the NIS Elements AR software. The mean fluorescence intensities over time were measured within the ROI using the time measurement module in NIS Elements software. Then, the ratio of GCaMP6s over mCherry mean fluorescence intensities within the ROI was calculated for each time point and averaged every second for quantitative comparison. For the analysis of frequency of Ca^2+^ transients, GCaMP6s/mCherry ratio was further processed by background elimination method arPLS (asymmetrically reweighted penalized least squares), based on penalized least squares for various spectra using Matlab software (MathWorks). The existing arPLS method was programmed according to a previous study (68). For the analysis of changes in baseline ciliary Ca^2+^ concentration before and after addition of drugs, the average GCaMP6s/mCherry ratios during the 4 min before and the 6 min after Shh stimulation (SAG addition) of the 10-min-total recording were compared.

Drug concentrations used to alter signaling in this and other methodological approaches were: 250 nM SAG (Calbiochem, #566660), 10-100 μM 1-[4-[(2,3,3-Trichloro-1-oxo-2-propen-1-yl)amino]phenyl]-5-(trifluoromethyl)-1*H*-pyrazole-4-carboxylic acid (Py3) (Tocris, #3751), 100 μM LaCl_3_, 10 μM SKF-96365 (Tocris, #1147), 10 μM XestosponginC (Calbiochem, #682160), 500 nM Cylopamine-KAAD (Calbiochem, #239804), 500 μM SQ22,536 (Sigma-Aldrich, # S153), 100 μM Vismodegib (Advanced ChemBlocks, #G-6429), and 25 μM GSK1702934A (Tocris, #6508). Control experiments consisted in adding saline alone or saline with treatment-equivalent concentrations (0.1% or 0.5%) of DMSO.

### Whole-Mount In Situ Hybridization.

The digoxigenin (DIG)-UTP-labeled antisense RNA was used as previously described (67). The following gene-specific primer sets were used to PCR amplify from the cDNAs and generate in situ probes: Sox2 (full length), forward 5′-ATGTACAGCATGATGGAGACCG, 5′-reverse TCACATGTGCGACAGAGGC; Ngn2 (full length), forward 5′-ATGGCTTCCAACATGGAAAGAG-3′, reverse 5′-CTAGTGGTACTGCATAAAGCAGT-3′; N-tubulin (amino acids 136-421), forward 5′-GACCCATTCTCTGGGTGGTG-3′, reverse 5′-CTCGGACACCAAGTCGTTCA-3′; Myt1 (amino acids 459-894), forward 5′-TCTGGCCAAGGAACTGGAGA-3′, reverse 5′-CTTTTCTTGGCACGTGGGC-3′. The labeled probes were detected with alkaline phosphatase-conjugated anti-DIG Fab fragments antibody (Roche, #11093274910, RRID:AB_514497) and visualized with the BM purple AP substrate (Roche Applied Science).

### RT-PCR and qRT-PCR.

RT-PCR was performed on cDNA synthesized (MMLV Reverse Transcriptase 1st-Strand cDNA Synthesis Kit, Epicentre) from total mRNA (SV Total RNA Isolation System, Promega) extracted from stage 1 whole embryo or neural tissues. –RT lane is the negative control of the RT-PCR on neural tube tissue RNA in the absence of a reverse transcriptase. The PCR primer sets are as follows: *trpc3*.S forward 5′-AGCAATGAGCTGGCAAAGTT-3′, reverse 5′-AATTGGGATGAGCCACAAAC-3′; *gapdh*.S forward 5′-TGCCAAGCGTGTCGTTATCT-3′, reverse 5′-TCTCCACAGCTTGCCTGATG-3′.

For qRT-PCR, embryos and tissue samples were resuspended in Trizol reagent (Invitrogen, cat. # 15596026) and stored at −80 °C. RNA was extracted with kit according to the manufacturer’s instructions (RNeasy Mini Kit, Qiagen, cat. # 74104), gDNA was eliminated (RapidOut DNA Removal Kit, Thermo Scientific, cat. # 00859896) and cDNA was made (High Capacity cDNA Reverse Transcription Kit, Applied Biosystems, cat. # 00890068) with standard protocols. Using this cDNA as template, qRT-PCR was performed with SYBR Green Universal Master Mix (Applied Biosystems, cat. # 2107118) in the Stratagene Mx3005 real-time PCR machine. RT-PCR program: 15 min 95 °C, 28 cycles of 45 s at 95 °C/30 s at 55 °C/ 30 s at 72 °C, 1 min at 95 °C, 30 s at 55 °C, and 30 s at 95 °C. Same primers mentioned above were used for *trpc3.S*. Primers for *odc* were, forward primer: GTCAATGATGGAGTGTATGGATC, reverse primer: TCCATTCCGCTCTCCTGAGCAC. *trpc3.S* PCR product: 236 bp, *odc* PCR product: 386 bp. All sequences are written from 5′ to 3′.

### TRPC3-Morpholino-Based Knockdown.

To knockdown TRPC3 expression during early embryonic development, splicing-blocking morpholino oligonucleotide (TRPC3-MO: 5′- ATTTCCCTTTTACGACTTACCTTGA -3′) (Gene Tools) targeting *X. laevis trpc3*.S exon/intron two splice junction was designed to interfere with *trpc3* mRNA splicing. A standard control morpholino oligonucleotide (Control-MO: 5′-CCTCTTACCTCAGTTACAATTTATA -3′) was injected as a control. TRPC3-MO or control-MO were injected into embryos at the two-cell stage. To confirm the effectiveness of TRPC3-MO knockdown, the following primers were designed to amplify the mature *trpc3* transcript encompassing exon 1-4: P1 forward, 5′- GGACAGTGGGTCCTACAGTGA -3′; P2 reverse, 5′- CAGCATCCACATCACCATTCAA -3′; P3 reverse, 5′- TGGTGCGATCCAGTAACCAA -3′.

### Immunocytochemistry and Whole-Mount Immunostaining.

*Xenopus* neural cultures were fixed in 4% paraformaldehyde in a cacodylate buffer (0.1 M sodium cacodylate, 0.1 M sucrose, pH 7.4) for 30 min and permeabilized with Triton X-100 (0.1%) for 10 min. The cells were incubated overnight at 4 °C with primary antibodies, followed by incubating with fluorescent secondary antibodies (Invitrogen) overnight at 4 °C. Fluorescent imaging was captured on a Nikon C1 or C2 confocal microscope. For whole-mount immunostaining, stage-14 through 26 embryos were fixed in 4% PFA for 4 h at 4 °C and bleached in 1:2 Dent’s fixative/H_2_O_2_ overnight at RT. Samples were washed, permeabilized in 1% Triton-X100, and incubated overnight at 4 °C with primary antibodies, followed by staining with fluorescent secondary antibodies at RT for 2 h, and finally clearing overnight in benzyl benzoate. Z-stack confocal images of embryos or neural tissue (100-μm-thick) were taken with a confocal microscope (Nikon C1 or C2), 10× or 20× objective, through approximately 30 to 100 steps (3- to 10-μm step) either longitudinally through a dorsoventral direction or transversely through an anteroposterior direction. Some embryos were cut into transverse segments to reveal more detailed structures. Primary antibodies used were TRPC3 (#3905, RRID:AB_741277; #ACC-016-GP, RRID:AB_2340963), Arl13b (#17711-1-AP, RRID:AB_2060867; #N295B/66, RRID:AB_2877361), GFP (#GFP-1020, RRID:AB_10000240; #TP401, RRID:AB_2313770), mCherry (#M11217, RRID:AB_2536611), Acetylated-tubulin (#sc-23950, RRID:AB_628409), Fox3 (#SIG-39860-100, RRID:AB_11220035), Sox2 (#AF2018, RRID:AB_355110), NCAM (#4d, RRID:AB_528389), CRMP4 (#orb5793, RRID:AB_10924203), Adenylate cyclase 3 (AC3; #orb5798, RRID:AB_10920137), and GPR161 (#13398-1-AP, RRID:AB_2113965).

### Immunohistochemistry of Neural Tissue Thin Sections.

Stage-22 to 23 embryos were fixed at 23 °C with 4% PFA for 10 min, and processed for immunostaining as previously described with modifications and by using standard protocols of paraffin embedding and sectioning (69). Incubations with primary and secondary antibodies were carried out overnight at 4 °C and for 2 h at 23 °C, respectively. Primary antibodies used were anti-TRPC3, 1:1000 (ProSci #3905) and acetylated a-tubulin, 1:1000 (SantaCruz; SC-23950). Antigen retrieval was performed by boiling samples in 0.05% citraconic anhydride, pH 7.4 for 10 min in water bath (70). Samples were permeabilized with PBST (0.5% Triton) for 1 h at 23 °C. Further processing starting with a 5% BSA in PBST (0.1% Triton) blocking step for 30 min was done using SNAP i.d. 2.0 System for immunohistochemistry (Millipore). Samples (12-μm-thick immunostained transverse sections) were imaged with a confocal microscope (Nikon A1), 60× objective through approximately 15 1-μm steps.

### BrdU Incorporation Assay.

Early neural plate (stage 12.5) embryos were microinjected at the blastocoel with 10 μM BrdU (Sigma-Aldrich, #B5002) and allowed to develop until mid-neural plate (stage 16) or early neural tube (stage 22) stages, when they were fixed and processed for whole-mount immunostaining as described above. For BrdU fluorescence immunostaining, processed embryos were equilibrated in DNase I buffer (40 mM Tris-HCl, pH 8.0, 10 mM MgSO_4_, 1 mM CaCl_2_) for 30 min at 37 °C, and treated with DNase I (0.1 unit/μL) for 2 h at 37 °C before subjecting to immunostaining with anti-BrdU antibody (#ab6326, RRID:AB_305426) as previously described (71).

### Quantitative Analysis of Whole-Mount Immunostained Neural Tissue.

Quantitative assessment of immunofluorescence staining for neuronal markers, Fox3, NCAM, and CRMP4 was performed by calculating the mean intensity in the 3D ROI encompassing the fluorescently labeled area defined by a surface object created with Imaris software (Bitplane).

Quantitative assessment of the number of cells immunopositive for Sox2 and BrdU was performed by using the Imaris ‘Spot’ function to detect nuclei objects filtered by object size, fluorescence intensity, and the built-in quality threshold.

Colocalization image was generated from 3D confocal images using Imaris built-in ‘Colocalization’ function and by creating a colocalization channel. For quantitative analysis of the colocalization between TRPC3 and primary ciliium marker immunolabeling, we performed ‘Spot’ detection to define two fluorescent signals and ‘Spot colocalization’ function in Imaris.

### CRISPR/Cas9-Induced TRPC3 Knockdown.

Single guide RNA (sgRNA) targeting *trpc3*.S was designed using the CRISPRscan website (72), and inDelphi model (73), which provide in silico predictions for mutational outcomes. The TRPC3 sgRNA (GTGACCATGATAAGGGACAA) was synthesized using the EnGen sgRNA synthesis kit (New England Biolabs). The sgRNA was complexed with Cas9 protein (PNA Bio, #CP02) at 300 mM KCl to form ribonucleoprotein (RNP) complexes and injected into embryos. The CrispantCal web tool (74) was used to calculate volumes corresponding to an optimal one-to-one molecular ratio of sgRNA to Cas9 in a CRISPR-Cas9 injection mix. To quantify the editing efficiency, genomic DNA was extracted from 5 edited embryos at early neural plate stages (stage 14) using DNeasy Blood & Tissue Kit (Qiagen). The edited locus of *trpc3* was amplified from genomic DNA using primers (forward, 5′- AGCCTAATGGGCCTTTTCTCTT -3′; reverse, 5′- AGCATCACCAATTCGTGCCA -3′) specific to the CRISPR/Cas9-targeted site for Sanger sequencing. The sequencing results were used for in silico analysis of the INDELs generated by the CRISPR/Cas9-mediated editing using Inference of CRISPR Edit analysis software (Synthego).

### Western Blot Assays.

Nuclear fraction was obtained from neural tube stage (stage 22) embryos previously incubated from early neural plate stages (stage 14) with 0.1% DMSO (vehicle), 100 μM Vismodegib, 25 μM GSK1702934A, or a mix of Vismodegib and GSK170 to assess expression of Sox2. Briefly, embryos were frozen in liquid nitrogen, stored at −80 °C, then homogenized in 25 mM Hepes pH 7.4, 50 mM NaCl, 2 mM EGTA, 5 mM MgCl_2_, and protease inhibitor cocktail (784115, Thermo Fisher Scientific) on ice for 30 min and centrifuged for 10 min at 1000 g. Nuclear pellets were resuspended in 1× protein loading buffer [125 mM Tris-HCl, pH 6.8, 4% SDS, 20% (w/v) glycerol, 0.005% Bromophenol Blue, 5% β-mercaptoethanol] and boiled for 5 min. Samples were run in 4 to 20% precast Tris-Glycine-eXtended PAGE gels (4561096, BioRad) and transferred to PVDF membrane. PVDF membrane was probed with anti-Sox2 goat polyclonal (#AF2018, RRID:AB_355110), 1:1,000 in 5% BSA at 4 °C, followed by incubation with horseradish peroxidase (HRP)-conjugated secondary antibody (#705-035-003, RRID:AB_2340390; 1:10,000) and visualized by Western Lightning Plus-ECL, Enhanced Chemiluminescence Substrate (NEL103E001, Perkin Elmer). PVDF membranes were stripped in 0.2 M glycine HCl buffer, pH 2.5, 0.05% Tween for 20 min and reprobed with 1:1,000 H2b antibody (#2934, RRID:AB_2295301) for nucleus-specific loading control in 5% milk powder, followed by incubation with horseradish peroxidase (HRP)-conjugated secondary antibody (#12-349, RRID:AB_390192, 1:10,000). Membranes were imaged with ChemiDoc-MP imaging instrument and optical density of bands of interest measured with associated software (Bio-Rad Laboratories).

### Quantification and Statistical Analysis.

Statistical analysis of the data was done with Prism software (Graphpad, Inc.). Normality test was performed in each set of data and then parametric (normally-distributed) or nonparametric statistical analysis was chosen. Paired tests were implemented in unilaterally manipulated embryos, when compared control and microinjected halves of neural tissue. The number of samples analyzed per group was more than five. Groups were considered statistically different when α < 0.05. Tests used included one-way ANOVA followed by Tukey’s multiple comparisons test, Wilcoxon matched pair signed rank test, nonparametric Kruskal–Wallis test followed by Dunn’s multiple comparisons test and Mann–Whitney U-test.

## Supplementary Material

Appendix 01 (PDF)

Dataset S01 (XLSX)

Movie S1.

Movie S2.

## Data Availability

All study data are included in the article and/or *SI Appendix*.

## References

[1] S. Ohnuma, W. A. Harris, Neurogenesis and the cell cycle. Neuron **40**, 199–208 (2003). 14556704 10.1016/s0896-6273(03)00632-9

[2] L. Lim, D. Mi, A. Llorca, O. Marín, Development and functional diversification of cortical interneurons. Neuron **100**, 294–313 (2018). 30359598 10.1016/j.neuron.2018.10.009 PMC6290988

[3] H. Okano, S. Temple, Cell types to order: Temporal specification of CNS stem cells. Curr. Opin. Neurobiol. **19**, 112–9 (2009). 19427192 10.1016/j.conb.2009.04.003

[4] P. Mill , Sonic hedgehog-dependent activation of Gli2 is essential for embryonic hair follicle development. Genes. Dev. **17**, 282–294 (2003). 12533516 10.1101/gad.1038103 PMC195973

[5] J. Cayuso, F. Ulloa, B. Cox, J. Briscoe, E. Marti, The Sonic hedgehog pathway independently controls the patterning, proliferation and survival of neuroepithelial cells by regulating Gli activity. Development **133**, 517–28 (2006). 16410413 10.1242/dev.02228

[6] A. Shkumatava, C. J. Neumann, Shh directs cell-cycle exit by activating p57Kip2 in the zebrafish retina. EMBO Rep. **6**, 563–569 (2005). 15891769 10.1038/sj.embor.7400416 PMC1369088

[7] E. Andersson , Identification of intrinsic determinants of midbrain dopamine neurons. Cell **124**, 393–405 (2006). 16439212 10.1016/j.cell.2005.10.037

[8] Y.-C. Ma , Regulation of motor neuron specification by phosphorylation of neurogenin 2. Neuron **58**, 65–77 (2008). 18400164 10.1016/j.neuron.2008.01.037 PMC2587148

[9] K. C. Corbit , Vertebrate smoothened functions at the primary cilium. Nature **437**, 1018–21 (2005). 16136078 10.1038/nature04117

[10] T. Caspary, C. E. Larkins, K. V. Anderson, The graded response to sonic hedgehog depends on cilia architecture. Dev. Cell **12**, 767–78 (2007). 17488627 10.1016/j.devcel.2007.03.004

[11] V. Singla, J. F. Reiter, The primary cilium as the cell’s antenna: Signaling at a sensory organelle. Science **313**, 629–633 (2006). 16888132 10.1126/science.1124534

[12] F. Hildebrandt, T. Benzing, N. Katsanis, Ciliopathies. N. Engl. J. Med. **364**, 1533–43 (2011). 21506742 10.1056/NEJMra1010172 PMC3640822

[13] A. Louvi, E. A. Grove, Cilia in the CNS: The quiet organelle claims center stage. Neuron **69**, 1046–1060 (2011). 21435552 10.1016/j.neuron.2011.03.002 PMC3070490

[14] K. Pal, S. Mukhopadhyay, Primary cilium and sonic hedgehog signaling during neural tube patterning: Role of GPCRs and second messengers: Neural tube patterning. Devel. Neurobio. **75**, 337–348 (2015). 10.1002/dneu.22193 24863049

[15] M. Bylund, E. Andersson, B. G. Novitch, J. Muhr, Vertebrate neurogenesis is counteracted by Sox1-3 activity. Nat. Neurosci. **6**, 1162–1168 (2003). 14517545 10.1038/nn1131

[16] V. Graham, J. Khudyakov, P. Ellis, L. Pevny, SOX2 functions to maintain neural progenitor identity. Neuron **39**, 749–65 (2003). 12948443 10.1016/s0896-6273(03)00497-5

[17] D. W. Hagey, J. Muhr, Sox2 acts in a dose-dependent fashion to regulate proliferation of cortical progenitors. Cell Rep. **9**, 1908–1920 (2014). 25482558 10.1016/j.celrep.2014.11.013

[18] O. V. Taranova , SOX2 is a dose-dependent regulator of retinal neural progenitor competence. Genes. Dev. **20**, 1187–1202 (2006). 16651659 10.1101/gad.1407906 PMC1472477

[19] L. Evsen, S. Sugahara, M. Uchikawa, H. Kondoh, D. K. Wu, Progression of neurogenesis in the inner ear requires inhibition of Sox2 transcription by neurogenin1 and neurod1. J. Neurosci. **33**, 3879–3890 (2013). 23447599 10.1523/JNEUROSCI.4030-12.2013 PMC3865497

[20] H. Takanaga , Gli2 is a novel regulator of sox2 expression in telencephalic neuroepithelial cells. Stem Cells **27**, 165–174 (2009). 18927476 10.1634/stemcells.2008-0580

[21] J. Ahlfeld , Sox2 requirement in sonic hedgehog-associated medulloblastoma. Cancer Res. **73**, 3796–3807 (2013). 23596255 10.1158/0008-5472.CAN-13-0238

[22] R. Favaro , Hippocampal development and neural stem cell maintenance require Sox2-dependent regulation of Shh. Nat. Neurosci. **12**, 1248–1256 (2009). 19734891 10.1038/nn.2397

[23] Y. H. Belgacem, L. N. Borodinsky, Inversion of sonic hedgehog action on its canonical pathway by electrical activity. Proc. Natl. Acad. Sci. U.S.A. **112**, 4140–4145 (2015). 25829542 10.1073/pnas.1419690112 PMC4386408

[24] N. Balaskas , Gene regulatory logic for reading the sonic hedgehog signaling gradient in the vertebrate neural tube. Cell **148**, 273–284 (2012). 22265416 10.1016/j.cell.2011.10.047 PMC3267043

[25] J. Lee, K. A. Platt, P. Censullo, A. Ruiz i Altaba, Gli1 is a target of sonic hedgehog that induces ventral neural tube development. Development **124**, 2537–2552 (1997). 9216996 10.1242/dev.124.13.2537

[26] Y. H. Belgacem, L. N. Borodinsky, Sonic hedgehog signaling is decoded by calcium spike activity in the developing spinal cord. Proc. Natl. Acad. Sci. U.S.A. **108**, 4482–4487 (2011). 21368195 10.1073/pnas.1018217108 PMC3060219

[27] L. N. Borodinsky , Activity-dependent homeostatic specification of transmitter expression in embryonic neurons. Nature **429**, 523–530 (2004). 15175743 10.1038/nature02518

[28] I. Swapna, L. N. Borodinsky, Interplay between electrical activity and bone morphogenetic protein signaling regulates spinal neuron differentiation. Proc. Natl. Acad. Sci. U.S.A. **109**, 16336–16341 (2012). 22991474 10.1073/pnas.1202818109 PMC3479614

[29] S. Malmersjo , Neural progenitors organize in small-world networks to promote cell proliferation. Proc. Natl. Acad. Sci. U.S.A. **110**, E1524–E1532 (2013). 23576737 10.1073/pnas.1220179110 PMC3631687

[30] K. A. Spencer , Growth at cold temperature increases the number of motor neurons to optimize locomotor function. Curr. Biol. **29**, 1787–1799.e5 (2019). 31130453 10.1016/j.cub.2019.04.072 PMC7501754

[31] N. C. Spitzer, Electrical activity in early neuronal development. Nature **444**, 707–712 (2006). 17151658 10.1038/nature05300

[32] S. Abdul-Wajid, H. Morales-Diaz, S. M. Khairallah, W. C. Smith, T-type calcium channel regulation of neural tube closure and EphrinA/EPHA Expression. Cell Rep. **13**, 829–839 (2015). 26489462 10.1016/j.celrep.2015.09.035 PMC4980084

[33] E. B. Sequerra, R. Goyal, P. A. Castro, J. B. Levin, L. N. Borodinsky, NMDA receptor signaling is important for neural tube formation and for preventing antiepileptic drug-induced neural tube defects. J. Neurosci. **38**, 4762–4773 (2018). 29712790 10.1523/JNEUROSCI.2634-17.2018 PMC5956989

[34] M. Delling, P. G. DeCaen, J. F. Doerner, S. Febvay, D. E. Clapham, Primary cilia are specialized calcium signalling organelles. Nature **504**, 311–314 (2013). 24336288 10.1038/nature12833 PMC4112737

[35] S. Su , Genetically encoded calcium indicator illuminates calcium dynamics in primary cilia. Nat. Methods **10**, 1105–1107 (2013). 24056873 10.1038/nmeth.2647 PMC3860264

[36] P. G. DeCaen, M. Delling, T. N. Vien, D. E. Clapham, Direct recording and molecular identification of the calcium channel of primary cilia. Nature **504**, 315–318 (2013). 24336289 10.1038/nature12832 PMC4073646

[37] D. Guo, C. Standley, K. Bellve, K. Fogarty, Z. Z. Bao, Protein kinase calpha and integrin-linked kinase mediate the negative axon guidance effects of sonic hedgehog. Mol. Cell Neurosci. **50**, 82–92 (2012). 22521536 10.1016/j.mcn.2012.03.008 PMC3383945

[38] X. Jin , Cilioplasm is a cellular compartment for calcium signaling in response to mechanical and chemical stimuli. Cell. Mol. Life Sci. **71**, 2165–2178 (2014). 24104765 10.1007/s00018-013-1483-1 PMC3981964

[39] C. R. Halaszovich, C. Zitt, E. Jungling, A. Luckhoff, Inhibition of TRP3 channels by lanthanides. Block from the cytosolic side of the plasma membrane. J. Biol. Chem. **275**, 37423–37428 (2000). 10970899 10.1074/jbc.M007010200

[40] J. E. Merritt , SK&F 96365, a novel inhibitor of receptor-mediated calcium entry. Biochem. J. **271**, 515–522 (1990). 2173565 10.1042/bj2710515 PMC1149585

[41] A. Singh, M. E. Hildebrand, E. Garcia, T. P. Snutch, The transient receptor potential channel antagonist SKF96365 is a potent blocker of low-voltage-activated T-type calcium channels. Br. J. Pharmacol. **160**, 1464–1475 (2010). 20590636 10.1111/j.1476-5381.2010.00786.x PMC2938817

[42] B. C. Bandyopadhyay , Apical localization of a functional TRPC3/TRPC6-Ca2+-signaling complex in polarized epithelial cells. J. Biol. Chem. **280**, 12908–12916 (2005). 15623527 10.1074/jbc.M410013200

[43] R. Rohatgi, W. J. Snell, The ciliary membrane. Curr. Opin. Cell Biol. **22**, 541–546 (2010). 20399632 10.1016/j.ceb.2010.03.010 PMC2910237

[44] A. M. Session , Genome evolution in the allotetraploid frog Xenopus laevis. Nature **538**, 336–343 (2016). 27762356 10.1038/nature19840 PMC5313049

[45] S. Kiyonaka , Selective and direct inhibition of TRPC3 channels underlies biological activities of a pyrazole compound. Proc. Natl. Acad. Sci. U.S.A. **106**, 5400–5405 (2009). 19289841 10.1073/pnas.0808793106 PMC2664023

[46] S. Mukhopadhyay , The ciliary G-protein-coupled receptor Gpr161 negatively regulates the sonic hedgehog pathway via cAMP signaling. Cell **152**, 210–23 (2013). 23332756 10.1016/j.cell.2012.12.026

[47] D. U. Mick , Proteomics of primary cilia by proximity labeling. Dev. Cell **35**, 497–512 (2015). 26585297 10.1016/j.devcel.2015.10.015 PMC4662609

[48] M. E. Truong , Vertebrate cells differentially interpret ciliary and extraciliary cAMP. Cell **184**, 2911–2926.e18 (2021). 33932338 10.1016/j.cell.2021.04.002 PMC8450001

[49] V. A. Bachmann , Gpr161 anchoring of PKA consolidates GPCR and cAMP signaling. Proc. Natl. Acad. Sci. U.S.A. **113**, 7786–7791 (2016). 27357676 10.1073/pnas.1608061113 PMC4948347

[50] S.-H. Sheu , A serotonergic axon-cilium synapse drives nuclear signaling to alter chromatin accessibility. Cell **185**, 3390–3407.e18 (2022). 36055200 10.1016/j.cell.2022.07.026 PMC9789380

[51] D. Klatt Shaw , Intracellular calcium mobilization is required for sonic hedgehog signaling. Dev. Cell **45**, 512–525.e5 (2018). 29754802 10.1016/j.devcel.2018.04.013 PMC6007892

[52] A. Li , Calcium oscillations coordinate feather mesenchymal cell movement by SHH dependent modulation of gap junction networks. Nat. Commun. **9**, 5377 (2018). 30560870 10.1038/s41467-018-07661-5 PMC6299091

[53] G. Boulay , Modulation of Ca(2+) entry by polypeptides of the inositol 1,4, 5-trisphosphate receptor (IP3R) that bind transient receptor potential (TRP): Evidence for roles of TRP and IP3R in store depletion-activated Ca(2+) entry. Proc. Natl. Acad. Sci. U.S.A. **96**, 14955–14960 (1999). 10611319 10.1073/pnas.96.26.14955 PMC24754

[54] E. Yildirim, B. T. Kawasaki, L. Birnbaumer, Molecular cloning of TRPC3a, an N-terminally extended, store-operated variant of the human C3 transient receptor potential channel. Proc. Natl. Acad. Sci. U.S.A. **102**, 3307–3311 (2005). 15728370 10.1073/pnas.0409908102 PMC552946

[55] T. Hofmann , Direct activation of human TRPC6 and TRPC3 channels by diacylglycerol. Nature **397**, 259–263 (1999). 9930701 10.1038/16711

[56] L. Vuolo, A. Herrera, B. Torroba, A. Menendez, S. Pons, Ciliary adenylyl cyclases control the hedgehog pathway. J. Cell. Sci. **128**, 2928–2937 (2015). 26092933 10.1242/jcs.172635

[57] B. Wang, J. F. Fallon, P. A. Beachy, Hedgehog-regulated processing of Gli3 produces an anterior/posterior repressor gradient in the developing vertebrate limb. Cell **100**, 423–434 (2000). 10693759 10.1016/s0092-8674(00)80678-9

[58] J. Y. Jiang, J. L. Falcone, S. Curci, A. M. Hofer, Direct visualization of cAMP signaling in primary cilia reveals up-regulation of ciliary GPCR activity following Hedgehog activation. Proc. Natl. Acad. Sci. U.S.A. **116**, 12066–12071 (2019). 31142652 10.1073/pnas.1819730116 PMC6575585

[59] L. V. Goodrich, L. Milenkovic, K. M. Higgins, M. P. Scott, Altered neural cell fates and medulloblastoma in mouse patched mutants. Science **277**, 1109–1113 (1997). 9262482 10.1126/science.277.5329.1109

[60] J. Taipale , Effects of oncogenic mutations in smoothened and patched can be reversed by cyclopamine. Nature **406**, 1005–1009 (2000). 10984056 10.1038/35023008

[61] Y.-G. Han, A. Alvarez-Buylla, Role of primary cilia in brain development and cancer. Curr. Opin. Neurobiol. **20**, 58–67 (2010). 20080044 10.1016/j.conb.2009.12.002 PMC2829308

[62] F. J. Swartling, M. Čančer, A. Frantz, H. Weishaupt, A. I. Persson, Deregulated proliferation and differentiation in brain tumors. Cell Tissue Res. **359**, 225–254 (2015). 25416506 10.1007/s00441-014-2046-y PMC4286433

[63] A. Maklad, M. Sedeeq, M. J. G. Milevskiy, I. Azimi, Calcium signalling in medulloblastoma: An in silico analysis of the expression of calcium regulating genes in patient samples. Genes **12**, 1329 (2021). 34573310 10.3390/genes12091329 PMC8468187

[64] R. Sutter , Cerebellar stem cells act as medulloblastoma-initiating cells in a mouse model and a neural stem cell signature characterizes a subset of human medulloblastomas. Oncogene **29**, 1845–1856 (2010). 20062081 10.1038/onc.2009.472

[65] R. J. Vanner , Quiescent Sox2+ cells drive hierarchical growth and relapse in sonic hedgehog subgroup medulloblastoma. Cancer Cell **26**, 33–47 (2014). 24954133 10.1016/j.ccr.2014.05.005 PMC4441014

[66] H. J. Selvadurai , Medulloblastoma arises from the persistence of a rare and transient Sox2+ granule neuron precursor. Cell Rep. **31**, 107511 (2020). 32294450 10.1016/j.celrep.2020.03.075

[67] S. Shim, J. Q. Zheng, G. L. Ming, A critical role for STIM1 in filopodial calcium entry and axon guidance. Mol. Brain **6**, 51 (2013). 24289807 10.1186/1756-6606-6-51 PMC3907062

[68] S.-J. Baek, A. Park, Y.-J. Ahn, J. Choo, Baseline correction using asymmetrically reweighted penalized least squares smoothing. Analyst **140**, 250–257 (2015). 25382860 10.1039/c4an01061b

[69] O. A. Balashova, O. Visina, L. N. Borodinsky, Folate receptor 1 is necessary for neural plate cell apical constriction during Xenopus neural tube formation. Development **144**, 1518–1530 (2017). 28255006 10.1242/dev.137315 PMC5399658

[70] S. Namimatsu, M. Ghazizadeh, Y. Sugisaki, Reversing the effects of formalin fixation with citraconic anhydride and heat: A universal antigen retrieval method. J. Histochem. Cytochem. **53**, 3–11 (2005). 15637333 10.1177/002215540505300102

[71] A. V. Tkatchenko, Whole-mount BrdU staining of proliferating cells by DNase treatment: Application to postnatal mammalian retina. BioTechniques **40**, 29–32 (2006). 16454036 10.2144/000112094

[72] M. A. Moreno-Mateos , CRISPRscan: Designing highly efficient sgRNAs for CRISPR-Cas9 targeting in vivo. Nat. Methods **12**, 982–988 (2015). 26322839 10.1038/nmeth.3543 PMC4589495

[73] M. W. Shen , Predictable and precise template-free CRISPR editing of pathogenic variants. Nature **563**, 646–651 (2018). 30405244 10.1038/s41586-018-0686-x PMC6517069

[74] A. Burger , Maximizing mutagenesis with solubilized CRISPR-Cas9 ribonucleoprotein complexes. Development **143**, 2025–2037 (2016). 27130213 10.1242/dev.134809

