# Supplementary material for: Calcium dynamics at the neural cell primary cilium regulate Hedgehog signaling–dependent neurogenesis in the embryonic neural tube
Source: Proc Natl Acad Sci U S A. 2023 May 30;120(23):e2220037120. doi: 10.1073/pnas.2220037120 (PMC10266006; doi:10.1073/pnas.2220037120)
Supplement: Supplementary file 1 — Appendix 01 (PDF) [file pnas.2220037120.sapp.pdf]

PNAS

Supporting Information for
Calcium dynamics at the neural cell primary cilium regulate Hedgehog signaling-dependent neurogenesis in the embryonic neural tube

Sangwoo Shim*, Raman Goyal, Alexios A. Panoutsopoulos, Olga A. Balashova, David Lee, Laura N. Borodinsky*

Laura Borodinsky and Sangwoo Shim
Email: lnborodinsky@ucdavis.edu; swshim@ucdavis.edu

This PDF file includes:

Figures S1 to S7

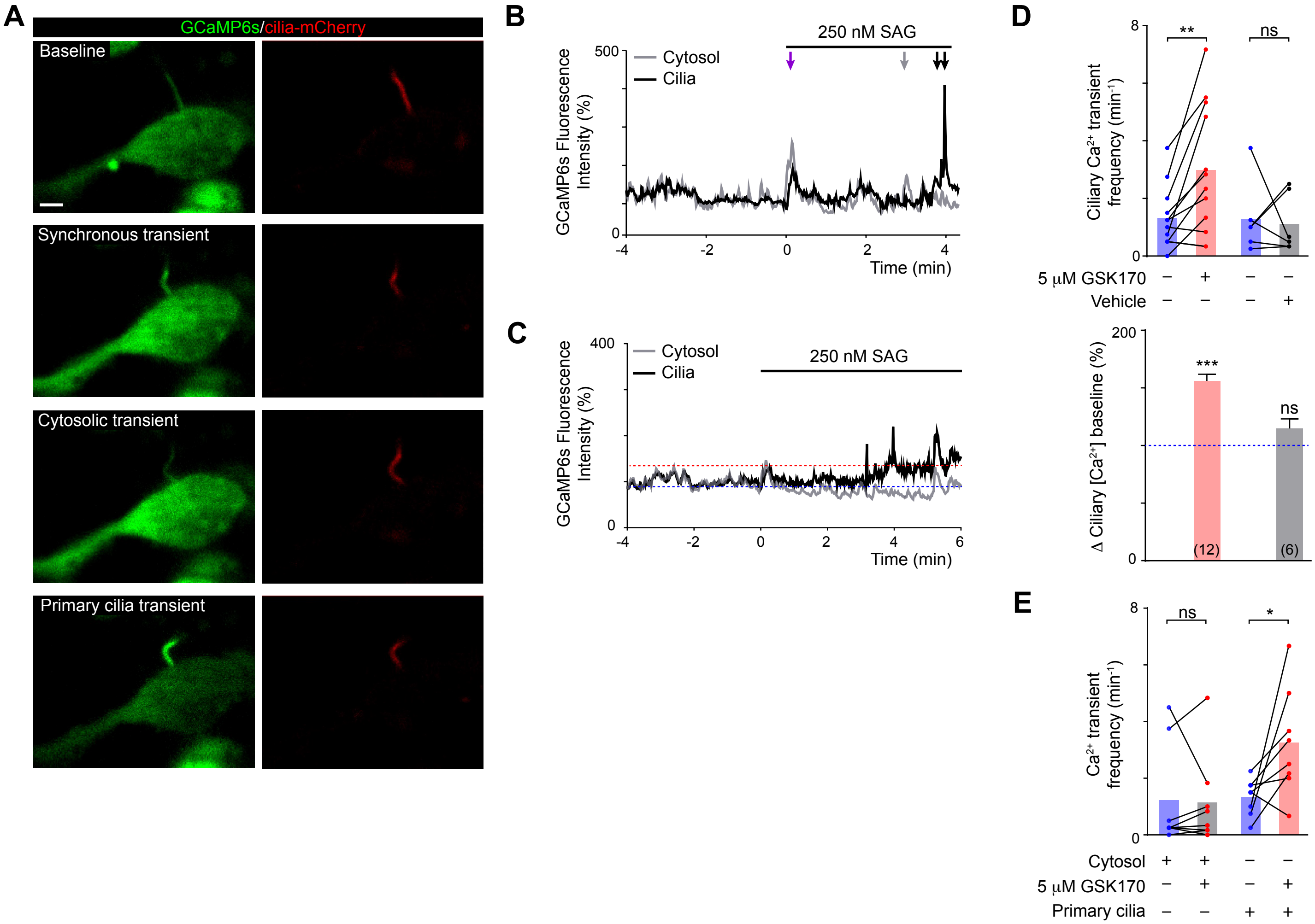
A
GCaMP6s/cilia-mCherry
Baseline
Synchronous transient
Cytosolic transient
Primary cilia transient
B
250 nM SAG
Cytosol
Cilia
GCaMP6s Fluorescence Intensity (%)
Time (min)
C
250 nM SAG
Cytosol
Cilia
GCaMP6s Fluorescence Intensity (%)
Time (min)
D
Ciliary Ca2+ transient frequency (min-1)
**
ns
5 μM GSK170
Vehicle
Δ Ciliary [Ca2+] baseline (%)
***
ns
(12)
(6)
E
Ca2+ transient frequency (min-1)
ns
*
Cytosol
5 μM GSK170
Primary cilia

Fig. S1. Simultaneous recording of whole-cell and ciliary Ca^{2+} dynamics. Dissociated cell cultures from wild type *Xenopus laevis* embryo neural tube (stage 23) expressing the ciliary Ca^{2+} reporter 5HT6-mCherry-GCaMP6s and the cytosolic Ca^{2+} reporter GCaMP6s were obtained and time-lapse imaged for recording GCaMP6s (green) and mCherry (red) fluorescence at the neuronal primary cilium and cytosol with an acquisition rate of 3.3-5 Hz for a total of 10 min before and after addition of 250 nM SAG (Smo agonist, *A*-*C*) or 5 μM GSK1702934A (GSK170, TRPC3 agonist, *D*). (*A*) Ca^{2+} transients can be synchronous in cytosol and primary cilium or restricted to one of these compartments. Images are examples of all the types of transients observed. Scale bar, 5 μm. (*B*) Example trace of Ca^{2+} activity (GCaMP6s/mCherry fluorescence intensity) at the neuronal primary cilium and cytosol before (- x axis) and after (+ x axis) addition (0 min) of 250 nM SAG to cultured neurons. (*C*) Average trace of changes in Ca^{2+} baseline (GCaMP6s/mCherry fluorescence intensity) at the neuronal primary cilium and cytosol before (- x axis) and after (+ x axis) addition (0 min) of 250 nM SAG to cultured neurons. Dotted lines represent relative ciliary Ca^{2+} baseline levels reached before (100%, blue) or after (red) SAG addition. (*D*-*E*) TRPC3 functionally localizes to the neuronal primary cilium. Paired data points in *D* and *E* connected with lines show Ca^{2+} transient frequency for individual primary cilia (*D*, *E*) or cytosol (*E*) before and after GSK170 (*D*, *E*) or vehicle (*D*) addition. Bar graph in *D* shows mean±SEM percent change in baseline GCaMP6s/mCherry ratio fluorescence intensity, representing change in baseline ciliary Ca^{2+} concentration, after addition of GSK170. N of cilia analyzed for each group are between parentheses. *p<0.05, **p<0.01, ***p<0.001, ns: not significant, Wilcoxon matched pairs signed rank test.

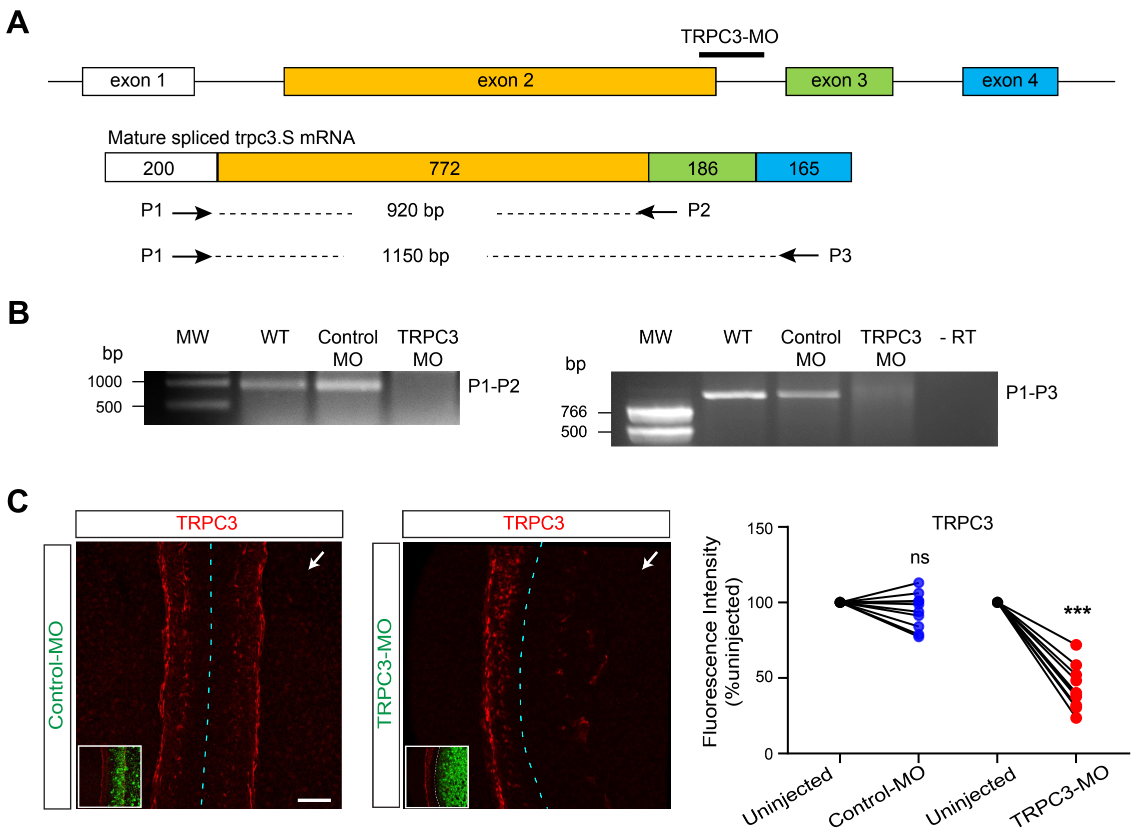

A
TRPC3-MO
exon 1
exon 2
exon 3
exon 4
Mature spliced trpc3.S mRNA
200
772
186
165
P1
920 bp
P2
P1
1150 bp
P3
B
MW
WT
Control MO
TRPC3 MO
bp
1000
500
P1-P2
MW
WT
Control MO
TRPC3 MO
- RT
bp
766
500
P1-P3
C
TRPC3
TRPC3
Control-MO
TRPC3-MO
TRPC3
150
100
50
0
ns
***
Fluorescence Intensity (%uninjected)
Uninjected
Control-MO
Uninjected
TRPC3-MO

Fig. S2. TRPC3 knockdown by splicing blocking TRPC3-MO. (*A*) Diagram of the genomic structure of the *trpc3*.S homeolog and mature spliced *trpc3*.S mRNA illustrating the position of the splice blocking morpholino targeting the splicing junction of exon 2/intron of *trpc3*.S mRNA, and gene-specific primers (P1, P2, P3) which are designed to monitor exon 2 skipping. (*B*, *C*) Two-cell stage embryos were bilaterally (*B*) or unilaterally (*C*) injected with 2.5 pmol TRPC3- or Standard Control-morpholino (TRPC3-MO or Control-MO) along with GFP mRNA (*C*) and processed for RT-PCR (*B*) or whole-mount immunostaining (*C*) when they reach early neural tube stages (stage 20). (*C*) Images are representative examples of the dorsal view of whole-mount immunostained neural tubes (stage 20) for TRPC3 protein. Insets show side expressing injected tracer (GFP immunostaining in green). Arrows indicate injected side. Dashed lines indicate midline. Scale bar, 50 μm. Graph shows data points of relative mean fluorescence intensity in 3D ROI in injected side compared with uninjected side in individual embryos analyzed using Imaris software. N≥10 embryos, ***$p<0.001$, ns: non-significant, Wilcoxon matched pairs signed rank test.

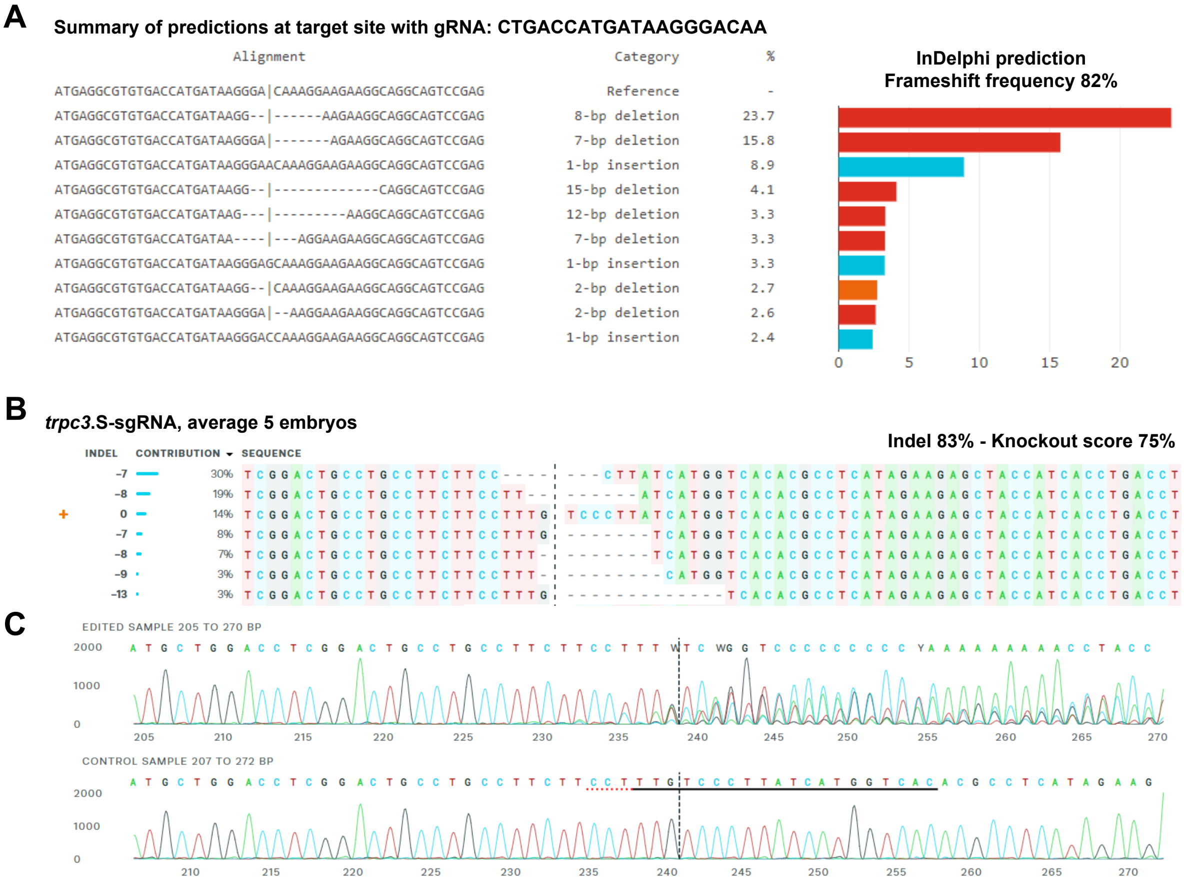
A
Summary of predictions at target site with gRNA: CTGACCATGATAAGGGACAA
Alignment
Category
%
ATGAGGCGTGTGACCATGATAAGGGA|CAAAGGAAGAAGGCAGGCAGTCCGAG Reference -
ATGAGGCGTGTGACCATGATAAGG--|------AAGAAGGCAGGCAGTCCGAG 8-bp deletion 23.7
ATGAGGCGTGTGACCATGATAAGGGA|-------AGAAGGCAGGCAGTCCGAG 7-bp deletion 15.8
ATGAGGCGTGTGACCATGATAAGGGAACAAAGGAAGAAGGCAGGCAGTCCGAG 1-bp insertion 8.9
ATGAGGCGTGTGACCATGATAAGG--|------------CAGGCAGTCCGAG 15-bp deletion 4.1
ATGAGGCGTGTGACCATGATAAG---|---------AAGGCAGGCAGTCCGAG 12-bp deletion 3.3
ATGAGGCGTGTGACCATGATAA----|---AGGAAGAAGGCAGGCAGTCCGAG 7-bp deletion 3.3
ATGAGGCGTGTGACCATGATAAGGGAGCAAAGGAAGAAGGCAGGCAGTCCGAG 1-bp insertion 3.3
ATGAGGCGTGTGACCATGATAAGG--|CAAAGGAAGAAGGCAGGCAGTCCGAG 2-bp deletion 2.7
ATGAGGCGTGTGACCATGATAAGGGA|--AAGGAAGAAGGCAGGCAGTCCGAG 2-bp deletion 2.6
ATGAGGCGTGTGACCATGATAAGGGACCAAAGGAAGAAGGCAGGCAGTCCGAG 1-bp insertion 2.4
InDelphi prediction
Frameshift frequency 82%
0
5
10
15
20
B
trpc3.S-sgRNA, average 5 embryos
Indel 83% - Knockout score 75%
INDEL
CONTRIBUTION
SEQUENCE
-7 30%
-8 19%
0 14%
-7 8%
-8 7%
-9 3%
-13 3%
C
EDITED SAMPLE 205 TO 270 BP
CONTROL SAMPLE 207 TO 272 BP
2000
1000
0

Fig. S3. Prediction and experimental outcomes of CRISPR/Cas9 gene editing against *trpc3*.S in *Xenopus* embryos. (*A*) InDelphi prediction of *trpc3*.S CRISPR/Cas9-mediated editing outcome. (*B*, *C*) Two-cell stage embryos were unilaterally injected with 1 ng Cas9 protein and 0.5 ng sgRNA against *trpc3*.S and processed for sequencing and ICE knockout analysis at stage 22. (*B*) Experimental assessment of CRISPR/Cas9-mediated *trpc3* gene deletion by ICE knockout analysis. The Cas9 cleavage site is indicated in vertical dashed line in *B* and *C*. Wild-type sequence is marked by + (left, 0). (*C*) Sanger sequencing chromatograms showing edited and control sequences around the region of the guide sequence as underlined with horizontal black line. PAM site is underlined with dotted red line.

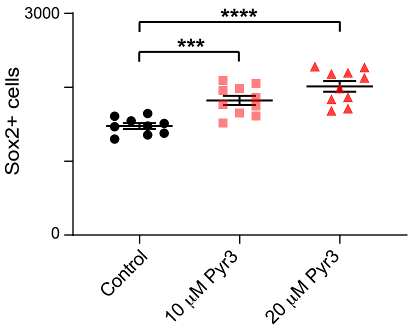

3000
0
Sox2+ cells
****
***
Control
10 μM Pyr3
20 μM Pyr3

Fig. S4. Pyr3 increases the number of Sox2+ cells in the developing neural tube in a concentration-dependent manner. Wild type embryos were grown until early neural plate stage (stage 14) when they were incubated with 10 or 20 μM Pyr3 (TRPC3 inhibitor), or vehicle only (0.1% DMSO, Control) for 7.75 h until they reached early neural tube stages (stage 23), when they were processed for whole-mount immunostaining for Sox2. Graph shows number of Sox2-expressing cells per embryo analyzed and mean±SEM. ***p<0.001, ****p<0.0001, Mann-Whitney U-test.

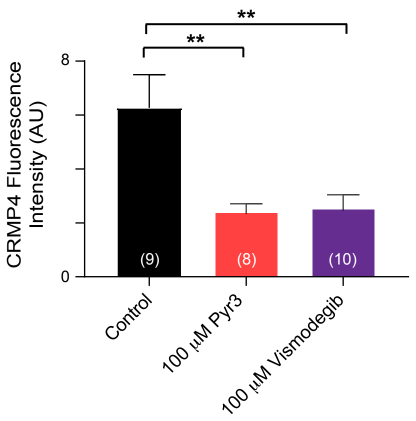

**
**
8
0
CRMP4 Fluorescence Intensity (AU)
(9)
(8)
(10)
Control
100 μM Pyr3
100 μM Vismodegib

Fig. S5. Inhibiting TRPC3 or Shh-Smo during neural progenitor-to-neuron transition decreases expression of neuronal differentiation marker CRMP4. Wild type embryos were grown until early neural plate stage (stage 14) when they were incubated with 100 μM Pyr3 (TRPC3 inhibitor), 100 μM vismodegib (Smo inhibitor), or vehicle only (0.1% DMSO, Control) for 7.75 h until they reached early neural tube stages (stage 23), when they were processed for whole-mount immunostaining. Graph shows mean±SEM fluorescence intensity of CRMP4 immunolabeling, N of embryos indicated in parentheses. **$p<0.01$, Mann-Whitney U-test.

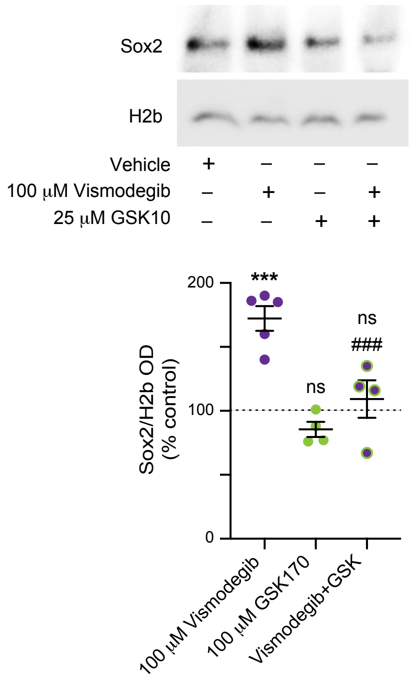

Sox2
H2b
Vehicle + – – –
100 μM Vismodegib – + – +
25 μM GSK10 – – + +
***
ns
ns
###
200
100
0
Sox2/H2b OD (% control)
100 μM Vismodegib
100 μM GSK170
Vismodegib+GSK

Fig. S6. Shh signaling regulates Sox2 protein levels by a TRPC3-dependent mechanism. Wild type embryos were grown until early neural plate stage (stage 14) when they were incubated with 100 μM Vismodegib (Smo inhibitor), 25 μM GSK170 (TRPC3 agonist), a mix of Vismodegib and GSK170, or vehicle only (0.1% DMSO, Control) until they reached early neural tube stages (stage 22) when they were processed for Western blot assays for comparison of Sox2 protein levels. Shown is a representative Western blot assay of the nuclear fraction enriched in Sox2 transcription factor. H2b was used as a nuclear fraction loading control. Graph shows individual experiment and mean±SEM percent of control (dashed line) ratios of Sox2/H2b band density for both proteins measured with BioRad imager software, $^{***}p<0.001$ and ns (not significant) compared with control (100%, dashed line), $^{\#\#\#}p<0.001$ compared with 100 mM Vismodegib, ordinary one-way ANOVA, Tukey's multiple comparisons test.

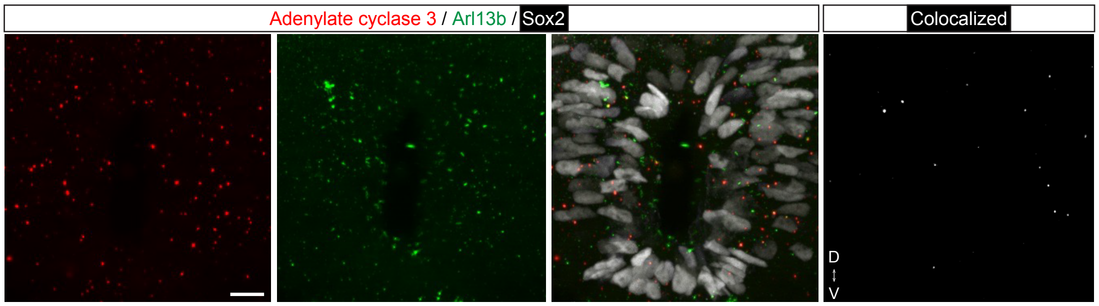
Adenylate cyclase 3 / Arl13b / Sox2
Colocalized
D
V

Fig. S7. Adenylate cyclase 3 is expressed in the developing neural tube and localizes to a subset of primary cilia. Transverse representative images of whole-mount early neural tube (stage 22) immunostained for adenylate cyclase 3 (AC3), primary cilia marker Arl13b and Sox2. Shown are maximum intensity projections (20X objective, 30 optical frames). The colocalized panel represents cilia labeled by both AC3 and Arl13b in a single optical frame.
